# Anticancer metallohelices: nanomolar potency and high selectivity†
†Electronic supplementary information (ESI) available: Experimental details, syntheses, biophysical analyses, antimicrobial, anticancer, toxicity, and mechanistic studies. See DOI: 10.1039/c5sc03677a



**DOI:** 10.1039/c5sc03677a

**Published:** 2015-10-26

**Authors:** Rebecca A. Kaner, Simon J. Allison, Alan D. Faulkner, Roger M. Phillips, David I. Roper, Samantha L. Shepherd, Daniel H. Simpson, Nicholas R. Waterfield, Peter Scott

**Affiliations:** a Department of Chemistry , University of Warwick , Coventry , CV4 7AL , UK . Email: peter.scott@warwick.ac.uk; b Institute of Advanced Study , University of Warwick , CV4 7HS , UK; c School of Applied Sciences , University of Huddersfield , Huddersfield , HD1 3DH , UK; d Warwick Medical School , University of Warwick , Coventry , CV4 7AL , UK; e School of Life Sciences , University of Warwick , Coventry , CV4 7AL , UK

## Abstract

A range of new helicate-like architectures have been prepared *via* highly diastereoselective self-assembly using readily accessible starting materials. Six pairs of enantiomers [Fe_2_L_3_]Cl_4_·*n*H_2_O (L = various bidentate ditopic ligands NN–NN) show very good water solubility and stability. Their activity against a range of cancer cell lines *in vitro* is structure-dependent and gives IC_50_ values as low as 40 nM. In an isogenic pair of HCT116 colorectal cancer cells, preferential activity was observed against cell lines that lack functional p53. Selectivity is also excellent, and against healthy human retinal pigment epithelial (ARPE19) and lung fibroblast (WI38) cells IC_50_ values are nearly three orders of magnitude higher. Cisplatin is unselective in the same tests. The compounds also appear to have low general toxicity in a number of models: there is little if any antimicrobial activity against methicillin-resistant *Staphylococcus aureus* and *Escherichia coli*; *Acanthamoeba polyphaga* is unaffected at 25 μg mL^–1^ (12.5 μM); *Manduca sexta* larvae showed clear evidence of systemic distribution of the drug, and rather than any observation of adverse effects they exhibited a significant mean weight gain *vs.* controls. Investigation of the mode of action revealed no significant interaction of the molecules with DNA, and stimulation of substantial cell death by apoptosis.

## Abstract 2

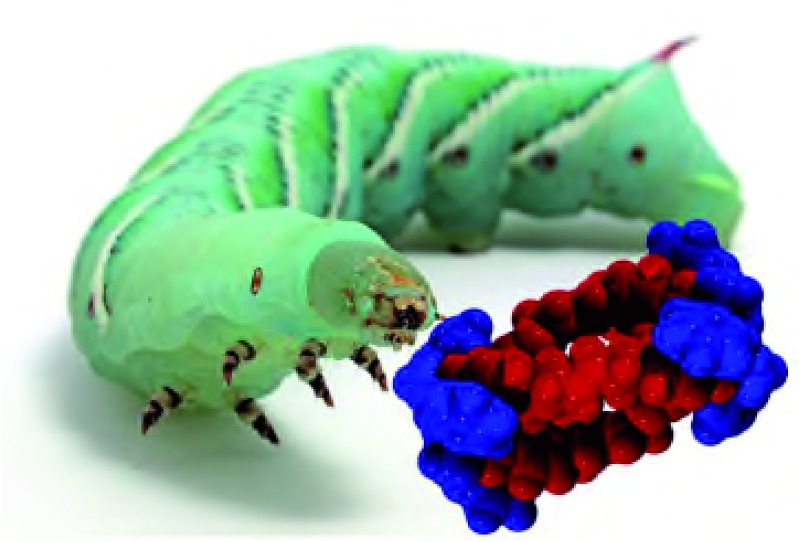
New optically pure helicate-like architectures are extremely active against cancer cell lines, with IC_50_ values as low as 40 nM, but nearly three orders of magnitude less active against healthy cells. There is also low toxicity to microbes and amoeba.

## Introduction

The main purpose of current anticancer therapies is to eradicate tumour cells without damaging overall patient health. However, side effects limit the dosage of chemotherapeutic drugs which may be safely applied, and as a result, cancer cells often remain. This leads to poor outcomes in the clinic and the evolution of drug-resistant tumours.^1^ Hence, while the potency of a drug is a very important consideration, drug selectivity towards cancer cells is key to ensuring both safety and effectiveness.^2^ While we might hope that more effective cancer chemotherapies would come from drugs designed to address specific biomolecular targets,^2^ this is far from uniformly the case.^3^ Such drugs may be too targeted since tumours can circumvent the blockade of a specific pathway by switching to another – so-called tumour plasticity.^4^ Compounds with polypharmacology (action against multiple targets) are thus currently of considerable interest to the pharmaceutical industry. This coincides with the resurgence of phenotypic drug discovery,^5–7^ where the targets of a drug are established after the observation of the useful biological effect. This strategy has led to a disproportionately high number of first-in-class drugs with novel mechanisms of action (1999–2008)^8,9^ The accompanying challenge for synthetic chemistry is to discover, perhaps without reference to some specific biomolecular target, new classes of drug candidates which are both potent and selective.

**Fig. 1:**
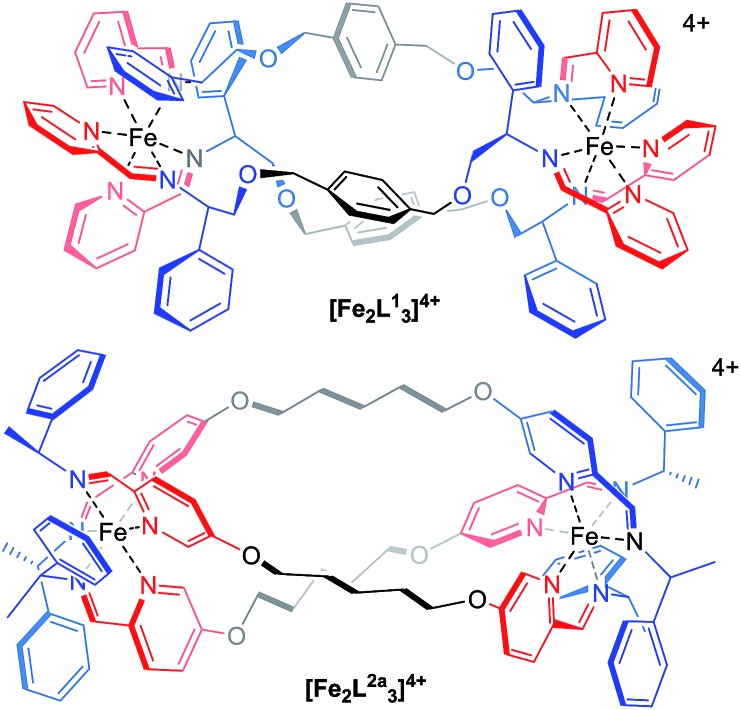
Structure of flexicates [Fe_2_ **L^1^**_3_]^4+^ and [Fe_2_ **L^A^**_3_]^4+^.^19^

Lehn recognized the potential of helicates in medicinal chemistry,^10^ and this was borne out in early studies, particularly in the area of cancer.^11–15^ We have argued,^16^ however, that in order for helicates to be capable of translation to the clinic a number of criteria need to be addressed: optical purity and stability, solubility and chemical stability in water, availability on a practical scale, and synthetic diversity. Our recent work has attempted to address these matters^17^ using a new strategy whereby the absolute configurations of individual metal centres are controlled^18^ and linked together to form the prototype helicate-like architectures of Fig. 1. Of these flexicates,^19^ [Fe_2_ **L^1^**_3_]^4+^ contains a diamine linker while [Fe_2_ **L^2a^**_3_]^4+^ is based on a dialdehyde.^19^ Promising results were reported in a number of disease areas,^16,19–21^ including good activity against a range of cancer cell lines.^20^ Here we report the discovery of a new series of highly potent (40 nM) anticancer compounds of the dialdehyde class related to [Fe_2_ **L^2a^**_3_]^4+^ that preferentially kill cancer cells that lack functional p53, are nearly three orders of magnitude less toxic to healthy human cell lines tested and have low toxicity to microbes, amoeba and caterpillar larvae.

While DNA does not appear to be the target, the compounds are triggering significant apoptotic cell death as part of their mode of action.

## Results

### Synthesis of ligands and Zn^II^ systems

**Fig. 2:**
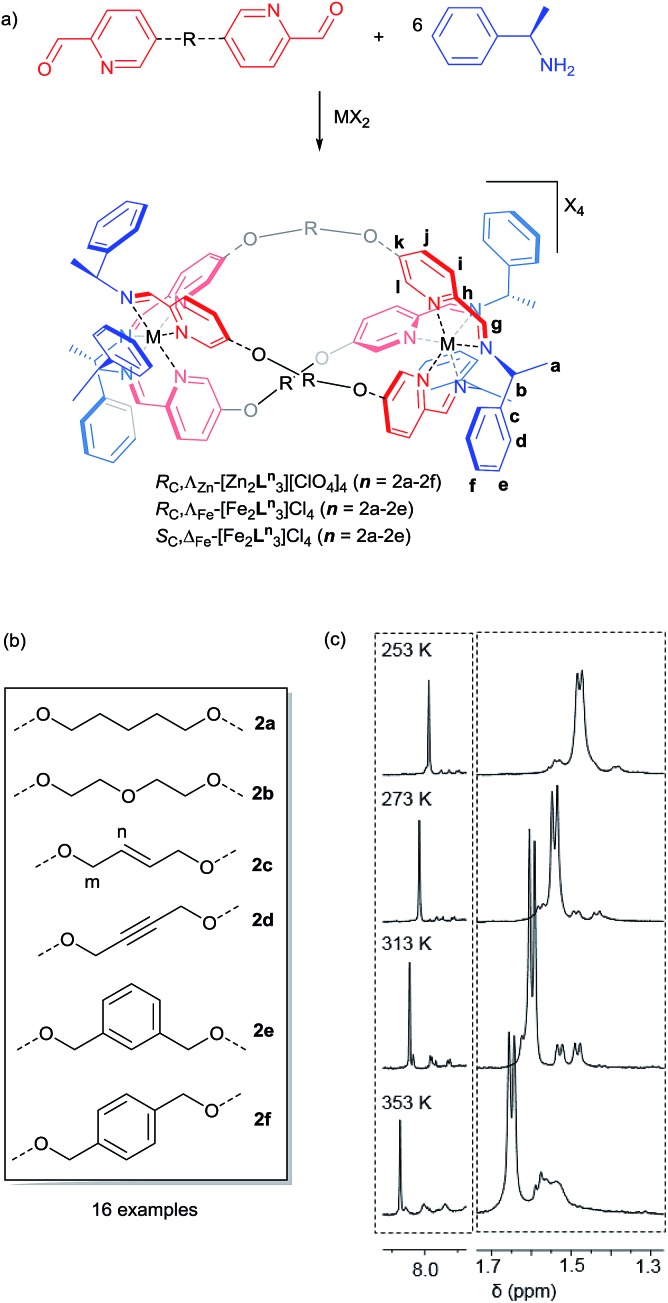
(a) Synthesis of new flexicates; (b) linking groups used in this study; (c) sections of ^1^H NMR spectra of Λ_Zn_-[Zn_2_ **L^2e^**_3_][ClO_4_]4·4H_2_O at 253–353 K in d^3^-acetonitrile showing the higher equilibrium population of a minor asymmetric conformer at higher temperatures.

The dialdehyde units of Fig. 2(a) and (b) include various linker rigidities and orientations designed to probe structural viability and biological activity. They were synthesized *via* simple etherifications of 5-hydoxypicolinaldehyde.^22^

Treatment with Zn(ClO_4_)_2_·6H_2_O and (*R*)-1-phenylethan-1-amine, in appropriate proportions, led to the rapid self-assembly of the bimetallic flexicates in acetonitrile solution at ambient temperature. For the majority of these new Zn^II^ complexes NMR spectra indicated that within the limits of the experiment single diastereomers were formed (*vide infra*).

The sole exception was the 1,3-phenylene system Λ_Zn_-[Zn_2_ **L^2e^**_3_][ClO_4_]_4_·4H_2_O, which gave more complex ^1^H NMR spectra [Fig. 2(c)]. At 253 K the phenethylamine methyl group doublet region 1.4–1.7 ppm contains one more intense doublet and two broader signals in the ratio *ca.* 10 : 1 : 1. The proportion of the minor species increases with temperature and the resonances sharpen somewhat, such that by 313 K two of the smaller doublets corresponding to the minor species are relatively sharp and resolved while a third overlaps with the main resonance. By 353 K the smaller peaks had again broadened considerably and the ratio of the two sets of resonances was *ca.* 10 : 9. The imine region (8.5–7.6 ppm) behaved in a corresponding manner (253 K, three peaks in ratio 10 : 1 : 1 : 1; 353 K, ratio 10 : 3 : 3 : 3). These observations are consistent with the presence of two species – one of high-symmetry and one low – in thermodynamic equilibrium (ratio *ca.* 1 : 0.3 at low temperature, increasing to almost 1 : 1 at high temperature) but with the involvement of other related conformers particularly at higher temperatures. The processes leading to the observed NMR behaviour may correspond to exchange between these conformers, or indeed between isostructural low symmetry species. While the spectra are not sufficiently well resolved to determine kinetic parameters, we sought to investigate this molecular system by computational means.

### Computational studies

**Fig. 3:**
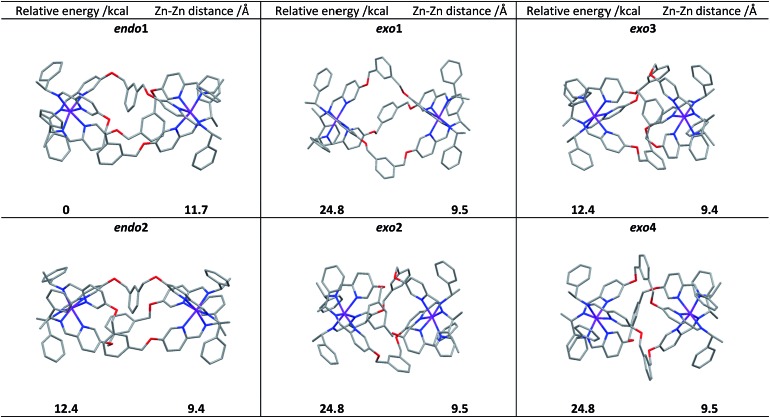
DFT calculated structures, relative energies (compared to *endo*1) and Zn–Zn distances of the six conformational isomers of Λ_Zn_-[Zn_2_ **L^2e^**_3_][ClO_4_]_4_.

Following extensive searching, six conformers of Λ_Zn_-[Zn_2_ **L^2e^**_3_]^4+^ were located and minimised [Fig. 3]. These fell into two classes: those where the three *m*-xylenyl groups were oriented away from the central cavity *i.e. exo*, and those where one such group was oriented *endo*. No conformers were observed in which two or three *m*-xylenyl groups were oriented into the cavity – this caused too much torsional and steric strain. Structure *endo*1 was found to be the lowest in energy, the next lowest being *endo*2 (*ca.* +5 kcal) which differs only in the fold of one of the linkers. For these structures the Zn–Zn distances are *ca.* 11.7 and 11.8 Å respectively. The structure *exo*1 (+7 kcal) has a large central cavity but a similar Zn–Zn distance (11.8 Å). The structure *exo*2 (+8 kcal) has a considerably shorter Zn–Zn distance at *ca.* 9.5 Å with accompanying concertinaed fold. Furthermore, higher energy conformers *exo*3 and *exo*4 differed principally in how the *m*-xylenyl groups folded towards each metal centre. Both were found to have a short Zn–Zn distance of 9.4 and 9.5 Å respectively.

While prediction of an equilibrium population from the above calculations is complicated by statistical and entropic contributions from the total number of possible structures and the differences in structural flexibility, the detection of two distinct structural classes is clearly consistent with observations in solution.

We propose that the species detected by NMR displaying high symmetry (*D*_3_) comprises *exo* conformations while the asymmetric (*C*_1_) species is *endo*. Examination of the structures indicates that the barrier to conversion within the *exo* or *endo* manifolds would be low since it would involve relatively simple concertina-type processes, but conversion between *exo* and *endo* conformations requires the rotation of the *m*-xylenyl linker through a strained, high energy transition state.

### Synthesis of water soluble compounds

**Fig. 4:**
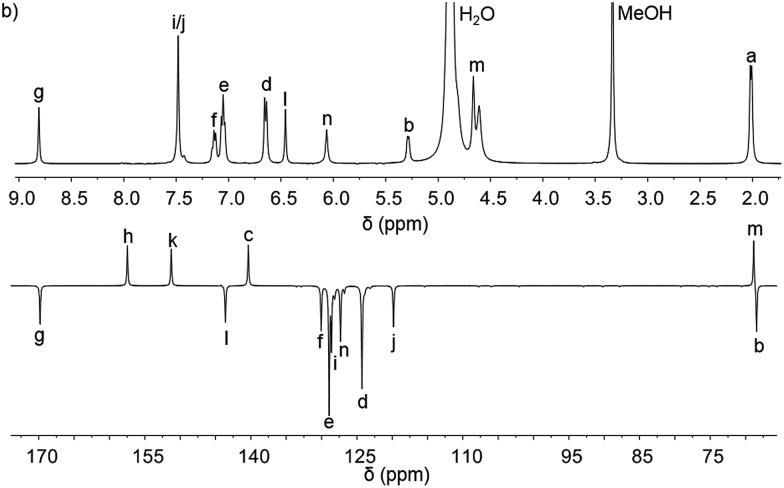
^1^H and ^13^C{^1^H}. NMR spectra of Λ_Fe_-[Fe_2_ **L^2c^**_3_]Cl_4_·9H_2_O at 298 K in d^4^-methanol; see Fig. 2 for key.

Pairs of water-soluble Fe^II^ flexicate enantiomers [Fe_2_ **L^*n*^**_3_]Cl_4_ (*n* = 2b–2e) were synthesised in high yield by heating the appropriate dialdehyde linker with either (*R*)- or (*S*)-1-phenylethan-1-amine and FeCl_2_ in methanol. ^1^H-NMR spectra were similar though slightly broader than the analogous Zn^II^ perchlorate complexes and are consistent, along with ^13^C-NMR [Fig. 4 and ESI†] and circular dichroism spectra (ESI†) with the presence of single, stable, non-racemising diastereomers in solution, although unsurprisingly [Fe_2_ **L^2e^**_3_]Cl_4_ exists as a similar mixture of conformers to the Zn analogue above. The complexes gave excellent electrospray mass spectrometry data *e.g.* Λ_Fe_-[Fe_2_ **L^2c^**_3_]Cl_4_ gave a strong peak at *m*/*z* 420.17 Da for the tetracationic ion. The formula weights of the panel of complexes, including levels of hydration, were determined by correlation of NMR, IR, thermogravimetric and elemental analyses (see ESI†). The *p*-xylenyl system [Fe_2_ **L^2f^**_3_]Cl_4_ displayed poor solubility in water and methanol and could not be fully characterised.

### Stability in aqueous media

Absorbance spectra indicated that little decomposition of the flexicates occurred in water at pH 7 over weeks, but half-lives for decomposition could readily be recorded in hydrochloric acid (0.2 M) *via* the 540 nm MLCT absorbance band of the complex. Even under such conditions, first order kinetic plots gave *t*_1/2_ values in the region 10–20 h. This very favourable aqueous stability of flexicates probably arises from the presence of extensive (hydrophobic) π-stacking.^23^

### Biological activity & selectivity

#### Cytotoxicity

**Fig. 5:**
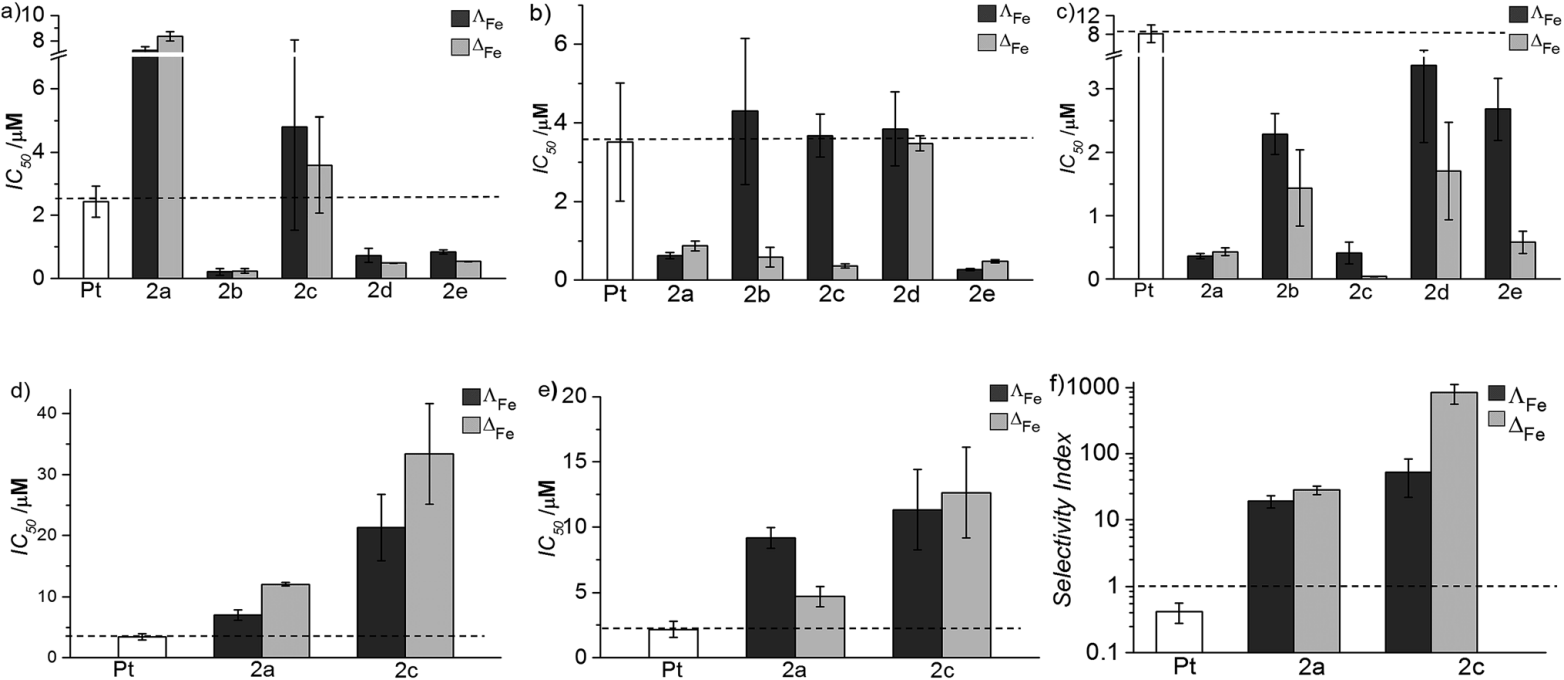
IC_50_ values of cisplatin (Pt, white) and flexicates [Fe_2_ **L^*n*^**_3_]Cl_4_ (*n* = 2a–2e) (Λ_Fe_ – dark grey, Δ_Fe_ – light grey) against (a) MDA-MB-468, (b) HCT116 p53^+/+^, (c) HCT116 p53^–/–^, (d) ARPE19 and (e) W138 cells over 96 h; dotted line highlights the relative sensitivity of test compounds compared to cisplatin, (f) *in vitro* selectivity index (IC_50_ ARPE19/IC_50_ HCT116 p53^–/–^) – broken line at selectivity index = 1 represents no difference in IC_50_ between tumour and normal cells – note log scale on *y* axis.

The activities of the new compounds and cisplatin were investigated in human tumour cell lines: (a) MDA-MB-468 (human epithelial breast adenocarcinoma);^24^ (b) HCT116 p53^+/+^ and (c) HCT116 p53^–/–^.^25^ The HCT116 p53^+/+^ and HCT116 p53^–/–^ cancer cells are human colorectal cancer cell lines that are genetically identical (isogenic) except for the presence or absence of functional p53.^25^ These were chosen to enable screening of the effects of p53 status as the loss of p53 function is common genetic event in patient tumours and is strongly associated with increased resistance to many conventional chemotherapeutic agents.^25,26^ In the cisplatin-sensitive (2.5 ± 0.5 μM) MDA-MB-468 cells, the new flexicates showed a range of activities [Fig. 5(a)], with enantiomers of the glycol-bridged [Fe_2_ **L^2b^**_3_]Cl_4_ being very potent (0.2 ± 0.1 μM); an order of magnitude more so than cisplatin. Against HCT116 p53^+/+^ cells [Fig. 5(b)] while cisplatin had a similar activity (3.5 ± 1.5 μM) as in the MDA-MB-468 cells, the flexicates were still more potent, with several examples having sub-micromolar activity, *e.g.* [Fe_2_ **L^2e^**_3_]Cl_4_ (0.4 ± 0.1 μM), and some significant enantiomeric differences were observed. Against HCT116 p53^–/–^ cells, cisplatin showed less activity (8.1 ± 1.8 μM) than for HCT116 p53^+/+^ cells (3.5 ± 1.5 μM) consistent with the increased resistance of cancer cells lacking p53 to many standard chemotherapeutic agents, while some of the flexicates were extremely active (2a annd 2c) with IC_50_ values in the nanomolar range *e.g.* Δ_Fe_-[Fe_2_ **L^2c^**_3_]Cl_4_ (40 ± 3 nM) [Fig. 5(c)]. Of particular note, flexicates Λ_Fe_-[Fe_2_ **L^2c^**_3_]Cl_4_ and Δ_Fe_-[Fe_2_ **L^2c^**_3_]Cl_4_ were both ∼9-fold more active against HCT116 p53^–/–^ cancer cells than their genetically identical p53^+/+^ counterparts [Fig. 5(b) and(c); see SEI].

#### Toxicity in healthy human cells

The most active flexicates in HCT116 p53^–/–^ cancer cells (2a and 2c), along with cisplatin, were investigated in human non-cancer retinal pigment epithelial cells (ARPE19)^27^ and normal lung fibroblasts (WI38) [Fig. 5(d)–(e)]. These are healthy human cells with a stable diploid karyotype which senesce after multiple passaging as is characteristic of non-cancer cells.^27^ In Fig. 5(f) we depict an *in vitro* selectivity index (SI) which compares the activity of these compounds in ARPE19 and HCT116 p53^–/–^ cells. While for cisplatin SI was found to be significantly less than 1, meaning that it is actually more toxic to these healthy cells than it is to the cancer cells, the flexicates tested gave SI substantially higher, and for Δ_Fe_-[Fe_2_ **L^2c^**_3_]Cl_4_ SI = 836 ± 280. This excellent selectivity prompted us to investigate the toxicity of the compounds against a number of organisms.

#### Toxicity to microbes

The compounds were screened against cultures of the gram-positive bacterium methicillin-resistant *Staphylococcus aureus*, USA300 JE2 (ref. 28 and 29) (MRSA) and the gram-negative *Escherichia coli*, TOP10 (*E. coli*).^30^ Kanamycin^30^ was used as a positive control.

The new flexicates had very modest antimicrobial activity (Table 1) or did not significantly inhibit microbial growth at concentrations well over 3 orders of magnitude higher than the IC_50_ values observed in cancer cells.

**Table 1:** MIC values for kanamycin and flexicates [Fe_2_ **L** ^***n***^_3_]Cl_4_ (*n* = 2a–2e) against MRSA and *E. coli*, over 20 h at 37 °C in Müller–Hinton broth. The approximate concentrations in μM are included for comparison with IC_50_ data from cancer cell line testing

Compound	MRSA MIC		*E. coli* MIC	
	(μg ml^–1^)	(μM)	(μg ml^–1^)	(μM)
Kanamycin	1	2	2	4
Λ_Fe_-[Fe_2_ **L^2a^**_3_]Cl_4_	64	35	>128	70
Λ_Fe_-[Fe_2_ **L^2b^**_3_]Cl_4_	128	70	>128	70
Λ_Fe_-[Fe_2_ **L^2c^**_3_]Cl_4_	128	70	>128	70
Λ_Fe_-[Fe_2_ **L^2d^**_3_]Cl_4_	>128	70	64	35
Λ_Fe_-[Fe_2_ **L^2e^**_3_]Cl_4_	64	35	128	70

#### Toxicity in amoebae and *M. sexta* larvae

We further tested the potential toxicity of these compounds using a single cell protist organism, the well-established amoeba model *Acanthamoeba polyphaga*. Concentrations of 2.5 μg mL^–1^ (1.25 μM) and 25 μg mL^–1^ (12.5 μM) showed no adverse effect on the amoeba development (see ESI†) also suggesting no broad-spectrum toxicity.

**Fig. 6:**
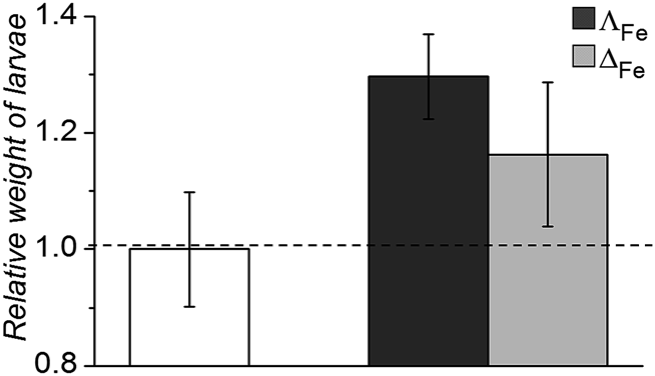
Relative weight of *Manduca sexta* larvae after treatment (7 d) with [Fe_2_ **L^2a^**_3_]Cl_4_ (Λ_Fe_ – dark grey, Δ_Fe_ – light grey) compared to an untreated control.

Oral and systemic toxicity of these flexicates was assessed in neonate larvae of *Manduca sexta*.^31^ Caterpillars which had ingested a solution of 25 μg mL^–1^ [12.5 μM], [Fe_2_ **L^2a^**_3_]Cl_4_ in artificial diet for 7 d [Fe_2_ **L^2a^**_3_]Cl_4_ showed comparable weight gain to controls suggesting no oral toxicity and no adverse effect on feeding behaviour (Fig. 6). Interestingly the larvae exhibited an increased mean weight gain of approximately 30% (*P* < 1). Also we noted that larvae that ingested the flexicate solutions turned a bright purple colour over the course of the assay, suggesting that these compounds were persisting in the insect and not being rapidly metabolized or excreted.

Systemic toxicity was further tested by injection of 50 μg (0.25 μM) of the compounds directly into the hemocoel of cohorts of 5^th^ instar larvae (*n* = 3). The cohorts were then allowed to continue feeding. Despite becoming purple, all larvae proceeded to develop into the pupal diapause stage as per the buffer control injections.

### Mode of action

The mode or modes of action of such a new and different system will require intensive investigation and is likely to involve multiple targets and pathways. Here, we describe two preliminary studies towards this end.

#### Denaturation of ct-DNA

We have previously concluded that the induction of DNA damage is not involved in the mode of action of earlier flexicates, despite particular examples binding in a cell free environment.^20,32,33^ We investigated the effect that the new flexicates had on the denaturation temperature (*T*_m_) of ct-DNA to screen for any indications of DNA binding.

Isolated ct-DNA (0.5 mg mL^–1^) was mixed with each flexicate (7.5 μM) in buffered conditions (10 mM tris, 1 mM EDTA at pH 7.0), to give 10 bases: 1 flexicate complex, and the absorbance at 260 nm between 25 °C and 90 °C was recorded (0.4 °C min^–1^). *T*_m_ for each experiment was calculated from the first derivative of a Boltzmann sigmoidal fit of the plot of absorbance *versus* temperature.

**Fig. 7:**
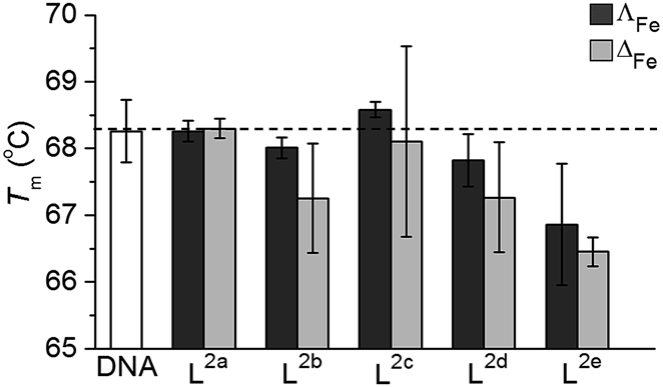
Effect on *T*_m_ of linear ct-DNA (DNA, white) from interactions [Fe_2_ **L^n^**_3_]Cl_4_ (*n* = 2a–2e) (Δ_Fe_ – light grey, Λ_Fe_ – dark grey) in 1 mM Trizma base (10 base pairs of DNA to 1 flexicate complex).

*T*_m_ of untreated ct-DNA (0.25 mg mL^–1^ in 10 mM tris, 1 mM EDTA at pH 7.0) was measured to be 68.3 ± 0.5 °C. Most of the new flexicates had no significant effect on the denaturation of ct-DNA (Fig. 7); the small (Δ*T ca.* 1 °C) reduction for **L^2e^** enantiomers can be ascribed to an electrostatic effect.^34^ We are therefore satisfied that DNA is unlikely to be the target of this panel of compounds.

#### Induction of cell death by apoptosis

The chemosensitivity observed could be due to cytostatic or cytotoxic effects, and cell death can occur by several different mechanisms. These include programmed cell death by apoptosis, inflammatory necrosis, autophagy or ‘self-eating’, necroptosis and pyroptosis.^35^ One of the hallmarks of cancers is the evasion of apoptosis, thus enabling the long-term survival and proliferation of cancer cells.^36^ We thus investigated whether the most active flexicates are stimulating apoptotic death in cancer cells as part their mode of action.

HCT116 p53^+/+^ cancer cells (24 h post-seeding) were incubated in fresh media containing flexicate or no flexicate (control) and were then analysed after 48 h for levels of apoptosis and necrosis. As cells start to undergo apoptosis, one of the first cellular changes is the externalisation of the membrane protein phosphatidylserine (PS). This can be detected and quantified by fluorescently labelled annexin V^37,38^ which can selectively bind externally exposed PS but is membrane-impermeable. This enables cells in the early stages of apoptosis to be distinguished from necrotic cells and cells in the late stages of apoptosis both of which have lost membrane integrity and will therefore also stain with the membrane-impermeable DNA stain propidium idodide.^39^

**Fig. 8:**
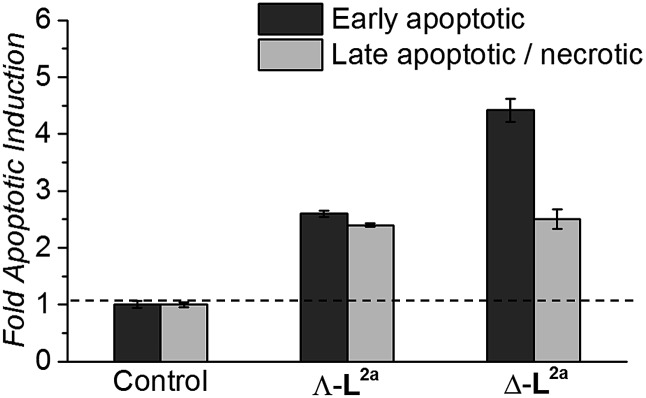
Induction of apoptosis in HCT116 p53^+/+^ cancer cells by 48 h treatment with the indicated flexicates. Fold change shown relative to basal apoptotic levels in untreated HCT116 control cells.

Flexicates Λ_Fe_-[Fe_2_ **L^2a^**_3_]Cl_4_ and Δ_Fe_-[Fe_2_ **L^2a^**_3_]Cl_4_ were tested and both induced significant levels of apoptosis that were ∼2.6 fold (Λ_Fe_-[Fe_2_ **L^2a^**_3_]Cl_4_) and ∼4.4 fold (Δ_Fe_-[Fe_2_ **L^2a^**_3_]Cl_4_) above background control levels in the HCT116 cancer cells at 48h (Fig. 8). A significant proportion of late apoptotic/necrotic cells were also detectable by 48 h, with levels ∼2.3–2.5 fold above background control levels (Fig. 8). These preliminary investigations indicate induction of apoptosis by these new flexicates as part of their mode of action.

## Experimental

### Synthesis

(*E*)-5,5′-(But-2-ene-1,4-diylbis(oxy))dipicolinaldehyde (0.13 g, 0.44 mmol) and (*R*)-1-phenylethan-1-amine (0.11 g, 0.88 mmol) were dissolved in acetonitrile (10 mL) with Zn^II^ perchlorate hexahydrate (0.11 g, 0.29 mmol) and the solution was stirred at ambient temperature for 20 h. Ethyl acetate was added drop-wise to cause precipitation of a white crystalline solid, Λ_Zn_-[Zn_2_ **L^2c^**_3_][ClO_4_]_4_·10H_2_O. Yield 0.214 g, 57%. ^1^H NMR (400 MHz, 298 K, CD_3_CN) *δ*_H_ 8.06 (6H, s, CHN), 7.49 (6H, dd, ^3^ *J*_HH_ = 8.5 Hz, ^4^ *J*_HH_ = 3.5 Hz), 7.36 (6H, d, ^3^ *J*_HH_ = 8.5 Hz), 7.14 (6H, d, ^3^ *J*_HH_ = 3.5 Hz), 7.09 (6H, t, ^3^ *J*_HH_ = 8.0 Hz), 6.95 (12H, t, ^3^ *J*_HH_ = 7.5 Hz), 6.64 (12H, d, ^3^ *J*_HH_ = 7.0 Hz, Ar), 6.12 (6H, m), 5.38 (6H, q, ^3^ *J*_HH_ = 6.5 Hz, CH), 4.64 (12H, s, CH_2_), 1.61 (18H, d, ^3^ *J*_HH_ = 6.5 Hz). ^13^C{^1^H} NMR (101 MHz, 298 K, CD_3_CN) *δ*_C_ 161.8 (CHN), 159.6, 142.0, 139.9, 139.4, 132.3 (Ar), 129.7 (CH), 129.4, 128.5, 126.4, 122.7 (Ar), 69.7 (CH_2_), 64.7 (CH), 23.6 (CH_3_). MS (ESI) *m*/*z* 411 [Zn_2_L_3_]^4+^. IR *ν* cm^–1^ 2976 w, 1570 m, 1316 m, 1225 m, 1082 s, 762 w, 703 m, 653 m. Elemental analysis found (calculated for C_96_H_96_Cl_4_N_12_O_22_Zn_2_·10H_2_O) % C 51.08 (51.88), H 4.82 (5.26), N 7.38 (7.56).

(*E*)-5,5′-(But-2-ene-1,4-diylbis(oxy))dipicolinaldehyde (0.1 g, 0.32 mmol) and (*R*)-1-phenylethan-1-amine (0.08 g, 0.63 mmol) were dissolved in methanol with Fe^II^ chloride (0.03 g, 0.21 mmol). The solution was stirred at reflux (75 °C) for 48 h and all volatiles were removed under reduced pressure to yield a dark purple solid, Λ_Fe_-[Fe_2_ **L^2c^**_3_]Cl_4_·9H_2_O. Yield 0.388 g, 97%. ^1^H NMR (400 MHz, 298 K, MeOD) *δ*_H_ 8.80 (6H, s, CHN), 7.47 (6H, s), 7.13 (6H, t, ^3^ *J*_HH_ = 7.0 Hz), 7.04 (12H, t, ^3^ *J*_HH_ = 7.0 Hz), 6.64 (12H, d, ^3^ *J*_HH_ = 7.0 Hz), 6.44 (6H, s), 6.05 (6H, s, Ar), 5.26 (6H, q, ^3^ *J*_HH_ = 6.0 Hz, CH), 4.65 (12H, s, CH_2_), 4.59 (6H, br s, CH), 1.99 (18H, d, ^3^ *J*_HH_ = 6.0 Hz, CH_3_). ^13^C{^1^H} NMR (101 MHz, 298 K, MeOD) *δ*_C_ 171.0 (CHN), 158.8, 152.5, 144.8, 141.7, 131.2, 130.1, 129.8, 128.5, 125.5 (Ar), 121.0 (CH), 70.0 (CH_2_), 69.6 (CH), 26.1 (CH_3_). MS (ESI) *m*/*z* 406 [Fe_2_L_3_]^4+^, 505 [L + H].IR *ν* cm^–1^ 3352 br s, 2970 br s, 1589 s, 1557 w, 1488 w, 1381 m, 1299 s, 1067 m, 1028 m, 760 m, 699 s, 562 w. Elemental analysis found (calculated for C_96_H_96_Cl_4_Fe_2_N_12_O_6_·9H_2_O) % C 59.92 (59.76), H 5.84 (5.96), N 8.63 (8.71).

### Molecular modelling

Models of a number of possible conformers of Λ_Zn_-[Zn_2_ **L^2e^**_3_][ClO_4_]_4_ were constructed and optimised. Starting points for geometry optimisations were taken from crystallographic data. Monometallic structures were first optimised using the B3LYP-D3(BJ)^40^ functional and the 6–31g* basis set, with convergence criteria of 0.0001 a.u. as implemented in the Firefly quantum chemistry package,^41^ which is partially based on the GAMESS(US) source code.^42^ Bimetallic systems were optimised using ligand field molecular mechanics (LFMM)^43^ as implemented in the DommiMOE program,^44^ before being annealed at 500 K for 1 ns prior to re-optimisation. Single point energy calculations of all structures were performed using the B3LYP-D3(BJ)^40^ functional and the deff2-TZVP basis set with energy convergence criteria of 0.0001 a.u. as implemented in the Firefly quantum chemistry package.^41^ The calculations were conducted by employing the RIJCOSX approximation with SCF convergence criteria set to ‘tight’, both of which are defined internally as part of the ORCA DFT quantum chemistry package.^45^ Where relevant, acetonitrile solvate correction was performed using the conductor-like screening model (COSMO)^46^ as implemented in ORCA.^45^

### Biological activity

MIC values were established using a macro broth dilution method in cation-adjusted Müller–Hinton (MH) broth. 96-well plates (200 μL of 128 μg mL^–1^, 64 μM) complex in MH broth, diluted 2^*n*^ μg mL^–1^, inoculated with each bacterial strain (10^3^ cfu mL^–1^) were sealed and growth was monitored over 20 h at 37 °C with an iEMS 96-well plate reader (see ESI†).

IC_50_ values were determined by incubating cells in 96-well plates (2.0 × 10^3^ cells per well) for 24 h at 37 °C, 5% CO_2_ prior to drug exposure. Compounds were added (100 μM to 5 nM in cell medium) for a further 96 h. 3-(4,5-Dimethylthiazol-1-yl)-2,5-diphenyl tetrazolium bromide solution (0.5 mg mL^–1^, 20 μL per well) was added for a final 4 h. Upon completion all solutions were aspirated, dimethyl sulfoxide (150 μl) was added and absorbance (540 nm) was recorded with a Thermo Scientific Multiskan EX microplate photometer.

Oral toxicity was established by feeding cohorts of *Manduca sexta* ^31^ one-day-old neonate larvae with each flexicate (25 μg mL^–1^ in artificial wheat germ diet) for 7 d at 28 °C and weighing to assess growth rate. Systemic toxicity assays^47^ were conducted by injecting an ethanol (70% v/v) swabbed region of first day fifth instar *M. sexta* larvae with each flexicate (0.5 mg mL^–1^ [0.25 μM] in PBS), before allowing them to continue feeding for 7 d at 28 °C, using physical stimulus to assess their status.

### Mode of action

Denaturation of ct-DNA was measured by mixing ct-DNA (0.5 mg mL^–1^, 7.5 × 10^–5^ per base, as determined by absorbance at 200 nm) with each complex (7.5 μM) in buffered conditions (10 mM tris, 1 mM EDTA at pH 7.0) to give 10 base: 1 complex. The absorbance at 260 nm as a function of temperature (every 1 °C, 25–90 °C) was measured in a 1 cm masked quartz cuvette at a rate of 0.4 °C min^–1^ and run in triplicate. *T*_m_ was calculated from the first derivative of a Boltzmann sigmoidal fit of the plot of absorbance at 260 nm against temperature for each complex.

Induction of apoptosis was determined by incubating HCT116 p53^+/+^ cells (5 × 10^5^ cells/flask, 10 mL complete RPMI-1640 medium) for 24 h at 37 °C in 5% CO_2_, before treating with each flexicate (20 μM in fresh complete media for 48 h) or fresh media containing no drug (control). The supernatant containing any non-adhered, floating cells was then collected and pooled with cells harvested by trypsinisation. This pooled single cell suspension was washed twice with PBS and incubated with propidium iodide and Annexin-V-FLUOS (Roche) to stain apoptotic cells in accordance with the manufacturer's instructions. The proportion of early stage apoptotic cells and late stage apoptotic/necrotic cells were then quantified by flow cytometry as previously described.^37,38^

## Conclusions

Our approach to metallohelix assembly has allowed us to generate a panel of biologically-compatible enantiomers incorporating various bridging groups. This was possible because in this so-called flexicate platform the stereochemistry of the metal complex units is predetermined very efficiently and largely independently of the bridges, and by a mechanism that also provides water-compatibility.^23,48^ In contrast, in a conventional “helication” approach the bridging units are structure-determining, so a mechanism of stereoselection would need to be designed for each example. A further advantage of the flexicate platform is beginning to emerge in that we may be able to develop asymmetric molecules from symmetric ligands *via* the kinds of conformational abnormalities caused by bridges that partially oppose the predetermined stereochemistry *e.g.* **L^2e^**. We have already shown that asymmetric (as opposed to merely chiral) optically pure assemblies are available using directional ligands.^49^ Further, this modular self-assembling system will allow us to probe the effects of peripheral functionality and lipophilicity.

The activity of these new assemblies against cancer cells is strongly dependent on structure, with a range of potencies from 30 μM to as low as 40 nM. The most active compound Δ_Fe_-[Fe_2_ **L^2c^**_3_]Cl_4_ shows a selectivity index (*versus* healthy cell lines) approaching 10^3^, demonstrating superiority over the clinically used anticancer drug cisplatin *in vitro* (SI < 1). This selectivity is substantiated in tests with various models; bacteria and amoeba exposed to high concentrations were essentially unaffected, and in *Manduca sexta* larvae, where the systemic stability of the drug is evidenced, there is arguably a pro-biotic effect *i.e.* the insects appear to thrive.

In respect of mechanism or mode of action, the lack of binding to DNA indicates that this is unlikely to be the general target in this panel. In fact only one early flexicate^19^ ([Fe_2_ **L^1^**_3_]^4+^, Fig. 1) in our growing library shows significant interactions with nucleic acids, and while there are fascinating selectivities with various motifs^19,32,33^ there is no DNA damage akin to that induced by *e.g.* platinum drugs and alkylators.^34,49^ Instead, relevant examples of protein interaction and enzyme inhibition have been characterised.^21,32^ To achieve drug safety and cancer selectivity, mechanistic classes which do not involve induction of DNA damage are attractive, and this may well be the source of the excellent selectivities we describe in this manuscript. Mode of action studies indicate that these compounds can induce substantial cell death by apoptosis independent of any DNA damage. Extensive studies are now required to understand how this complex process, normally subverted in cancers, is induced by these compounds. The above observations of remarkable selectivity alongside very high potency and large enantiomeric differences are however all consistent with a subtle mechanism involving the targeting of oncogenic drivers.
