# Supplementary material for: Anticancer metallohelices: nanomolar potency and high selectivity
Source: Chem Sci. 2015 Oct 26;7(2):951–8. doi: 10.1039/c5sc03677a (PMC5530816; doi:10.1039/c5sc03677a)
Supplement: Supplementary file 1 [file SC-007-C5SC03677A-s001.pdf]

Electronic Supplementary Material (ESI) for Chemical Science.
This journal is © The Royal Society of Chemistry 2015

Anticancer metallohelices; nanomolar potency and high selectivity

Rebecca A. Kaner, Simon J. Allison, Alan D. Faulkner, Roger M. Phillips, David I. Roper, Samantha L. Shepherd, Daniel H. Simpson, Nicholas R. Waterfield, and Peter Scott

Contents

Synthetic procedures 2

Solvents and chemicals 2

Equipment and instrumentation 2

Dipicolinaldehyde units 2

Zinc(II) flexicates 7

Water soluble iron(II) flexicates 11

Biophysical analysis of iron(II) flexicates 17

Cirular dichroism 17

Thermogravimetric analysis 17

Aqueous stability 19

Antimicrobial activity 21

Anticancer activity 22

Systemic and oral toxicity 23

Denaturation of ct-DNA 24

Induction of Apoptosis 25

References 26

Synthetic procedures

Solvents and chemicals

All solvents and chemicals purchased from commercial sources (Sigma-Aldrich, Acros, Fisher Scientific, Alfa Aesar or Invitrogen) were used without further purification unless otherwise stated. When necessary acetonitrile was dried by heating to reflux for 3 d under dinitrogen over calcium, degassed before use and stored in glass ampoules under argon. Deuterated solvents were purchased from Sigma-Aldrich or Cambridge Isotope Laboratories and pre-dried over molecular sieves (3A for methanol, dimethyl sulfoxide and acetonitrile; 4A for chloroform), for 24 h prior to use.

Equipment and instrumentation

Where appropriate, reactions were carried out under argon using a dual manifold argon/vacuum line and standard Schlenk techniques or an MBraun dry box. All glassware and cannulae for these techniques were stored in an oven at > 375 K.

NMR spectra were recorded on Bruker Spectrospin DPX-300/400 or Bruker AV II DRX-500 spectrometers. Routine NMR assignments were confirmed by ^{1}H-^{1}H (COSY) and ^{13}C-^{1}H (HMQC) correlation experiments where necessary. The spectra were internally referenced using the residual protio solvent ($CDCl_3$, CD_3CN etc.) resonance relative to tetramethylsilane (δ = 0 ppm). ESI mass spectra were recorded in a methanol-water mix (80:20) on either an Agilent Technologies 1260 Infinity spectrometer or a Bruker Daltonics MicroTOF spectrometer. Infra-red spectra were measured using a Bruker Alpha-P FTIR spectrometer. Elemental analyses were performed by Medac Ltd. Chobham, Surrey GU24, 8JB, UK or Warwick Analytical Service, Coventry, CV4 7EZ.

5-hydroxypicolinaldehyde,[1] 5,5'-(pentane-1,5-diylbis(oxy))dipicolinaldehyde,[2] bis(4-(bromomethyl) phenyl)methane[3], 1,4-dibromobut-2-yne[4], Λ_{Zn}-[Zn_2**L**$^{1a}_3$][ClO_4]$_4$,[5] Λ_{Zn}-[Zn_2**L**$^{2a}_3$][ClO_4]$_4$,[5] Λ_{Fe}-[Fe_2**L**$^{1a}_3$]Cl_4,[5] Δ_{Fe}-[Fe_2**L**$^{1a}_3$]Cl_4,[5] Λ_{Fe}-[Fe_2**L**$^{2a}_3$]Cl_4,[5] and Δ_{Fe}-[Fe_2**L**$^{2a}_3$]Cl_4[5] were synthesised following known methods and used in the following procedures.

Dipicolinaldehyde units

5,5'-(butane-1,4-diylbis(oxy))dipicolinaldehyde

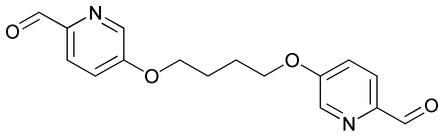

5-hydroxypicolinaldehyde (0.54 g, 4.4 mol) was dissolved in acetonitrile (50 ml). Potassium carbonate (0.64 g, 4.6 mol) followed by 1,4-dibromobutane (0.62 g, 2.7 mol) were added and the solution was stirred at reflux (80°C) for 16 h. The reaction mixture was filtered through a silica plug and the solvent was removed under reduced pressure. The crude product was dissolved in dichloromethane (50 ml), dried over sodium sulphate, filtered and the solvent was removed under reduced pressure to give a pale brown solid.

Yield 0.512 g, 84%.

^{1}H NMR (300 MHz, 298 K, $CDCl_3$) δ_H 9.93 (2H, s, CHO), 8.37 (2H, d, $^3J_{HH}$ = 2.5 Hz), 7.89 (2H, d, $^3J_{HH}$ = 9.0 Hz), 7.26 (2H, dd, $^3J_{HH}$ =9.0 Hz, $^4J_{HH}$ = 2.5 Hz, Ar), 4.15 (4H, m), 2.03 (4H, m, CH_2).

^{13}C{^{1}H} NMR (75 MHz, 298 K, $CDCl_3$) δ_C 192.1 (CHO), 158.4, 146.4, 138.8, 123.53, 120.6 (Ar), 68.3, 25.8 (CH_2).

MS (ESI) m/z 301 $[M+H]^+$, 323 $[M+Na]^+$

IR υ cm^{-1} 2824 w, 1700 m, 1568 m, 1306 m, 1205 s, 964 m, 831 m, 604 s.

Elemental analysis found (calculated for $C_{16}H_{16}N_2O_4$.½H_2O) % C 62.48 (62.13), H 5.59 (5.54), N 8.99 (9.06).

5,5'-(hexane-1,6-diylbis(oxy))dipicolinaldehyde

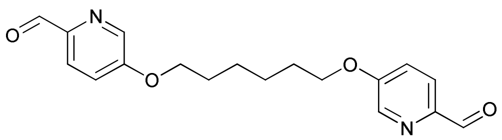

Synthesised using the procedure described for 5,5'-(butane-1,4-diylbis(oxy))dipicolinaldehyde substituting 1,4-dibromobutane for 1,6-dibromohexane.

Yield 0.406 g, 61%.

^{1}H NMR (300 MHz, 298 K, $CDCl_3$) δ_H 9.98 (2H, s, CHO), 8.41 (2H, d, $^3J_{HH}$ = 2.5 Hz), 7.93 (2H, d, $^3J_{HH}$ = 8.0 Hz), 7.26 (2H, dd, $^3J_{HH}$ = 9.0 Hz, $^4J_{HH}$ = 2.5 Hz, Py), 4.12 (4H, t, $^3J_{HH}$ = 6.0 Hz), 1.90 (4H, m), 1.58 (4H, m, CH_2).

^{13}C{^{1}H} NMR (75 MHz, 298 K, $CDCl_3$) δ_C 192.2 (CHO), 158.6, 146.4, 138.9, 123.54, 120.6 (Ar), 68.7, 29.0, 25.8 (CH_2).

MS (ESI) m/z 329 $[M+H]^+$, 351 $[M+Na]^+$

IR υ cm^{-1} 2951 w, 1700 m, 1567 s, 1315 s, 1208 m, 1011 m, 851 m, 656 s.

Elemental analysis found (calculated for $C_{18}H_{20}N_2O_4$.¼H_2O) % C 64.72 (64.95), H 6.39 (6.40), N 8.46 (8.09).

1-bromo-2-(2-bromoethoxy)ethane

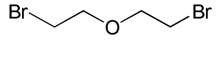

Triphenylphosphine (25 g, 94 mmol) was suspended in dry acetonitrile (20 ml) and cooled to 0°C using an ice-water bath. Bromine (5 ml, 94 mmol) was added dropwise followed by diethylene glycol (5 ml, 47 mmol). The reaction was stirred at reflux (80°C) for 18 h under dinitrogen. The solvent was removed

under reduced pressure and the residue was taken up in diethyl ether (150 ml). The solution was then filtered and the solvent was removed under reduced pressure to give the crude product as a yellow liquid. This was purified by Kügelrohr distillation to give a clear liquid (b.p. 95°C under high vacuum).

Yield 8.21 g, 76%.

^{1}H NMR (400 MHz, 298 K, $CDCl_3$) δ_H 3.83 (4H, t, $^{3}J_{HH}$ = 6.0 Hz, 3.48 (4H, t, $^{3}J_{HH}$ = 6.0 Hz, CH_2).

^{13}C-NMR (101 MHz, $CDCl_3$): δ 71.0, 30.2 (CH_2).

MS (ESI) m/z 233 $[M+H]^+$

IR υ cm^{-1}: 2966 w, 2856 w, 1739 m, 1438 w, 1421 m, 1361 w, 1279 m, 1226 w, 1111 s, 1030 m, 1005 m, 948 m, 726 m, 691 m, 663 m.

Elemental analysis found (calculated for $C_4H_8Br_2O$) % C 20.78 (20.72), H 3.49 (3.48).

5,5'-(2,2'-oxybis(ethane-2,1-diyl)bis(oxy))dipicolinaldehyde

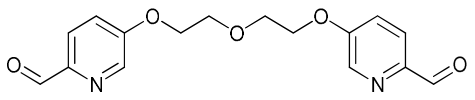

Synthesised using the procedure described for 5,5'-(butane-1,4-diylbis(oxy))dipicolinaldehyde, substituting 1,4-dibromobutane for 1-bromo-2-(2-bromoethoxy)ethane.

Yield 1.02 g, 75%.

^{1}H NMR (400 MHz, 298 K, CD_3CN): δ 9.99 (2H, s, CHO), 8.45 (2H, d, $^{3}J_{HH}$ = 2.5 Hz), 7.96 (2H, d, $^{3}J_{HH}$ = 8.5), 7.33 (2H, dd, $^{3}J_{HH}$ = 8.5 Hz, $^{4}J_{HH}$ = 2.5 Hz, Py), 4.30 (4H, t, $^{3}J_{HH}$ = 4.5 Hz), 4.00 (4H, t, $^{3}J_{HH}$ = 4.5 Hz, CH_2).

$^{13}C\{^{1}H\}$ NMR (75 MHz, 298 K,CD_3CN) δ 193.0 (CHO), 165.6, 163.6, 139.9, 124.1, 121.9 (Py), 70.1, 69.3 (CH_2).

MS (ESI) m/z 339 $[M+Na]^+$

IR υ cm^{-1} 3650 w, 2963 m, 1695 m, 1573 m, 1491 m, 1456 w, 1278 m, 1258 s, 1221 m, 1093 s, 1045 s, 1010 s, 947 m, 923 m, 842 m, 795 s, 765 m, 720 m, 696 m, 663 s.

Elemental analysis found (calculated for $C_{16}H_{16}N_2O_5$) % C 60.47 (60.76), H 5.05 (5.10), N 9.14 (8.85).

(E)-5,5'-(but-2-ene-1,4-diylbis(oxy))dipicolinaldehyde

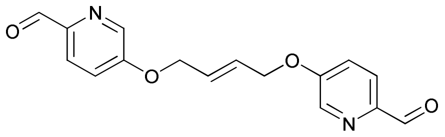

Synthesised using the procedure described for 5,5'-(butane-1,4-diylbis(oxy))dipicolinaldehyde, substituting 1,4-dibromobutane for 1,4-trans-dibromobut-2-ene.

Yield 0.323 g, 53%.

^{1}H NMR (300 MHz, 298 K, CD_3CN) δ_C 9.93 (2H, s, CHO), 8.46 (2H, d, $^{3}J_{HH}$ = 2.5 Hz), 7.91 (2H, d, $^{3}J_{HH}$ = 8.0 Hz), 7.47 (2H, dd, $^{3}J_{HH}$ = 8.0 Hz, $^{4}J_{HH}$ = 2.5 hz, Py), 6.16 (2H, m CH), 4.80 (4H, m, CH_2).

^{13}C{^{1}H} NMR (75 MHz, 298 K, CD_3CN) δ_C 193.0 (CHO), 165.4, 142.3, 140.0, 129.2, 124.1, 122.1 (Py), 69.1 (CH_2).

MS (ESI) m/z 321 [M+Na]$^{+}$

IR υ cm^{-1} 2844 w, 1710 m, 1566 m, 1273 s, 1121 s, 803 m, 609 m.

Elemental analysis found (calculated for $C_{16}H_{14}N_2O_4.½H_2O$) % C 62.85 (62.54), H 4.59 (4.92), N 9.11 (9.12).

5,5'-(but-2-yne-1,4-diylbis(oxy))dipicolinaldehyde

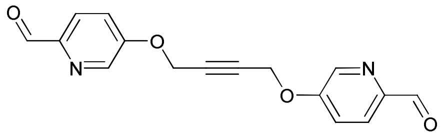

Synthesised using the procedure described for 5,5'-(butane-1,4-diylbis(oxy))dipicolinaldehyde, substituting 1,4-dibromobutane for 1,4-dibromobut-2-yne.

Yield 0.208 g, 60 %.

^{1}H NMR (400 MHz, 298 K, CD_3CN) δ_H 9.88 (2H, s, CHO), 8.40 (2H, d, $^{3}J_{HH}$ = 3.0 Hz), 7.80 (2H, d, $^{3}J_{HH}$ = 8.5 Hz), 7.40 (2H, dd, 1H, $^{3}J_{HH}$ = 8.5 Hz, $^{4}J_{HH}$ = 3.0 Hz, Py), 4.94 (4H, s, CH_2).

^{13}C{^{1}H} NMR (101 MHz, 298 K, CD_3CN) δ_C 192.8 (CHO), 160.1, 139.8, 134.5, 123.6, 122.2 (Py), 69.4, 57.1 (CH_2).

MS (ESI) m/z 297 [M+H]$^{+}$, 319 [M+Na]$^{+}$

IR υ cm^{-1} 2854 w, 1699 m, 1568 s, 1307 m, 1199 s, 998 m, 824 m, 611 s.

Elemental analysis found (calculated for $C_{16}H_{12}N_2O_4.½H_2O$) % C 62.85 (62.95), H 3.95 (4.29), N 9.11 (9.18).

5,5'-(1,3-phenylenebis(methylene))bis(oxy)dipicolinaldehyde

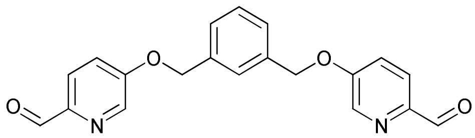

5-hydroxypicolinaldehyde (0.5 g, 4.1 mol) was dissolved in dimethylformamide (20 ml). Potassium carbonate (0.57 g 4.3 mol) followed by 1,3-bis(bromomethyl)benzene (0.55 g, 2.1 mol) were added and the solution was stirred at reflux (100°C) for 4 h. After removing the solvent under reduced pressure the crude material was dissolved in dichloromethane (100 ml), washed with sodium hydroxide solution (1M, 3 × 100 ml) and brine (3 × 100 ml), dried over sodium sulphate and the solvent was removed. This was then taken up in acetonitrile, filtered and the solvent was removed and a white solid was recovered following a hot hexane extraction.

Yield 0.615 g, 87%.

^{1}H NMR (300 MHz, 298 K, CD_3CN) δ_H 9.69 (2H, s, CHO), 8.27 (2H, d, $^{3}J_{HH}$ = 2.5 Hz), 7.68 (2H, d, $^{3}J_{HH}$ = 8.5 Hz), 7.38 (2H, s), 7.24 (4H, m, Ar), 5.05 (4H, s, CH_2).

^{13}C{^{1}H} NMR (75 MHz, 298 K, CD_3CN) δ_C 193.0 (CHO), 159.2, 142.6, 140.1, 137.6, 130.1, 128.9, 128.2, 124.1, 122.2 (Ar), 71.2 (OCH_2).

MS (ESI) m/z 347 $[M\text{-}H]^-$, 349 $[M+H]^+$, 371 $[M+Na]^+$, 383 $[M+Cl]^-$

IR υ cm^{-1} 3049 w, 2820 w, 1700 s, 1570 s, 1210 s, 1156 s, 1012 m, 838 m, 795 m, 616 m.

Elemental analysis found (calculated for $C_{20}H_{16}N_2O_4$) % C 68.45 (68.96), H 4.40 (4.63), N 8.23 (8.04).

5,5'-(1,4-phenylenebis(methylene))bis(oxy)dipicolinaldehyde

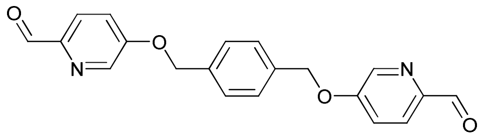

Synthesised using the procedure described for 5,5'-(butane-1,4-diylbis(oxy))dipicolinaldehyde, substituting 1,4-dibromobutane for 1,4-bis(bromomethyl)benzene.

Yield 0.636 g, 90%.

^{1}H NMR (400 MHz, 298 K, $CDCl_3$) δ_H 9.76 (2H, s, CHO), 8.27 (2H, d, $^{3}J_{HH}$ = 3.0 Hz), 7.72 (2H, d, $^{3}J_{HH}$ = 9.0 Hz), 7.26 (2H, s), 7.14 (2H, dd, $^{3}J_{HH}$ = 8.5 Hz, $^{4}J_{HH}$ = 2.5 Hz), 7.03 (2H, s, Ar), 4.99 (4H, s, CH_2).

^{13}C{^{1}H} NMR (101 MHz, 298 K, $CDCl_3$) δ_C 192.0 (CHO), 158.1, 146.6, 139.0, 135.7, 128.0, 123.3, 121.1 (Ar), 70.2 (OCH_2).

MS (ESI) m/z 371 $[M+Na]^+$

IR υ cm^{-1} 2821 w, 1698 m, 1569 s, 1312 m, 1206 s, 1010 m, 848 m, 602 m.

Elemental analysis found (calculated for $C_{20}H_{16}N_2O_4.¼H_2O$ % C 67.85 (68.08), H, 4.67 (4.71), N 8.41 (7.94).

Zinc(II) flexicates

Λ_{Zn}-[$Zn_2L^{2b}{}_3$][ClO_4]$_4$

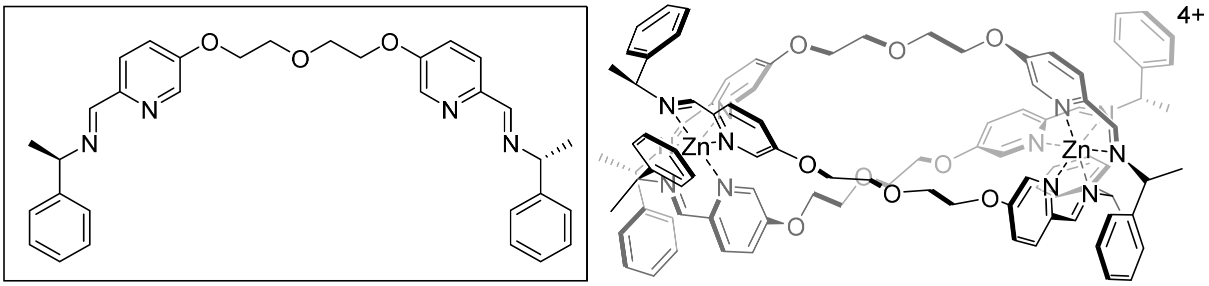

5,5'-(2,2'-oxybis(ethane-2,1-diyl)bis(oxy))dipicolinaldehyde (0.13 g, 0.44 mmol) and (*R*)-1-phenylethan-1-amine (0.11 g, 0.88 mmol) were stirred in acetonitrile (10 ml) for 1 h. Zinc (II) perchlorate hexahydrate (0.11 g, 0.29 mmol) was added and the solution was stirred at ambient temperature for 20 h ethyl acetate was added drop-wise to cause precipitation of a white crystalline solid.

Yield 0.390 g, 78%.

^{1}H NMR (400 MHz, 298 K, CD_3CN) δ_H 8.09 (6H, s, CHN), 7.49 (6H, dd, $^3J_{HH}$ = 8.5 Hz, $^4J_{HH}$ = 2.5 Hz), 7.41 (6H, d, $^3J_{HH}$ = 8.5 Hz), 7.06 (6H, t, $^3J_{HH}$ = 7.5 Hz), 6.96 (12H, t, $^3J_{HH}$ = 7.5 Hz), 6.91 (6H, d, $^4J_{HH}$ = 2.5 Hz), 6.67 (12H, d, $^3J_{HH}$ = 7.5 Hz, Ar), 5.35 (6H, q, $^3J_{HH}$ = 6.5 Hz, CH), 4.11-4.01 (12H, m), 3.85-3.74 (12H, m, OCH_2), 1.57 (18H, d, $^3J_{HH}$ = 6.5 Hz, CH_3).

^{13}C{^{1}H} NMR (101 MHz, 298 K, CD3CN) δ_C 161.6 (CHN), 156.0, 157.8, 154.2, 143.9, 138.2, 132.1, 129.6, 128.6, 126.6, 124.5 (Ar), 70.1, 70.0 (CH_2), 64.8 (CH), 23.7 (CH_3).

MS (ESI) m/z 523 [L+H]$^+$, 545 [L+Na]$^+$

IR υ cm^{-1} 2935 w, 1570 m, 1267 m, 1082 s, 651 w, 622 m.

Elemental analysis found (calculated for $C_{96}H_{96}Cl_4N_{12}O_{22}Zn_2.11H_2O$) % C 51.08 (51.46), H 4.82 (5.31), N 7.38 (7.50).

Λ_{Zn}-[Zn_2L^{2c}$_3$][ClO_4]$_4$

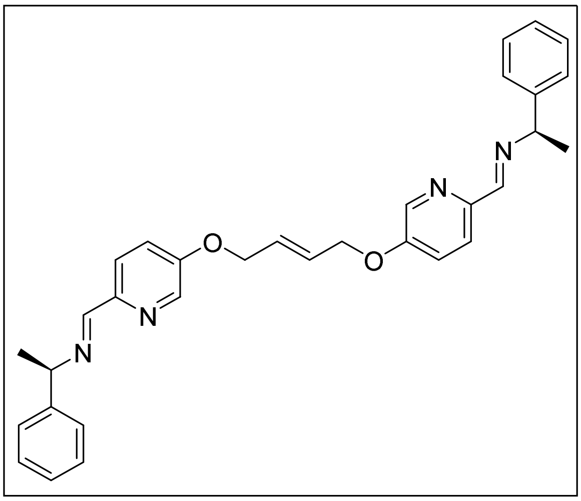

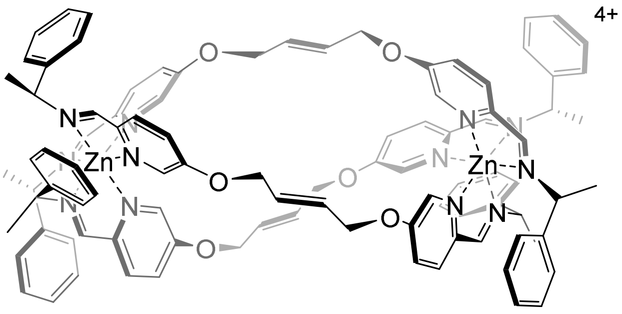

Λ_{Zn}-[Zn_2**L^{2c}**$_3$][ClO_4]$_4$ was synthesised using the procedure described for Λ_{Zn}-[Zn_2**L^{2b}**$_3$][ClO_4]$_4$, substituting 5,5'-(2,2'-oxybis(ethane-2,1-diyl)bis(oxy))dipicolinaldehyde for (E)-5,5'-(but-2-ene-1,4-diylbis(oxy))dipicolinaldehyde.

Yield 0.214 g, 61%.

^{1}H NMR (400 MHz, 298 K, CD_3CN) δ_H 8.06 (6H, s, CHN), 7.49 (6H, dd, $^3J_{HH}$ = 8.5 Hz, $^4J_{HH}$ = 3.5 Hz), 7.36 (6H, d, $^3J_{HH}$ = 8.5 Hz), 7.14 (6H, d, $^3J_{HH}$ = 3.5 Hz), 7.09 (6H, t, $^3J_{HH}$ = 8.0 Hz), 6.95 (12H, t, $^3J_{HH}$ = 7.5 Hz), 6.64 (12H, d, $^3J_{HH}$ = 7.0 Hz, Ar), 6.12 (6H, m), 5.38 (6H, q, $^3J_{HH}$ = 6.5 Hz, CH), 4.64 (12H, s, CH_2), 1.61 (18H, d, $^3J_{HH}$ = 6.5 Hz).

^{13}C{^{1}H} NMR (101 MHz, 298 K, CD_3CN) δ_C 161.8 (CHN), 159.6, 142.0, 139.9, 139.4, 132.3 (Ar), 129.7 (CH), 129.4, 128.5, 126.4, 122.7 (Ar), 69.7 (CH_2), 64.7 (CH), 23.6 (CH_3).

MS (ESI) m/z 411 [Zn_2L_3]$^{4+}$.

IR υ cm^{-1} 2976 w, 1570 m, 1316 m, 1225 m, 1082 s, 762 w, 703 m, 653 m.

Elemental analysis found (calculated for $C_{96}H_{96}Cl_4N_{12}O_{22}Zn_2.10H_2O$) % C 51.08 (51.88), H 4.82 (5.26), N 7.38 (7.56).

Λ_{Zn}-[$Zn_2$$L^{2d}$$_3$][$ClO_4$]$_4$

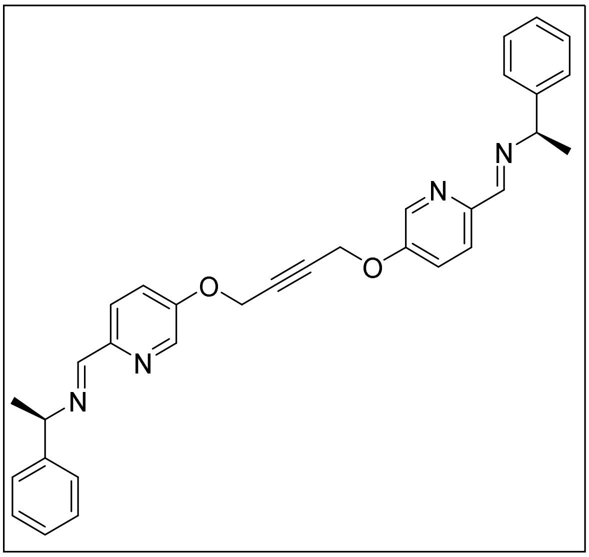

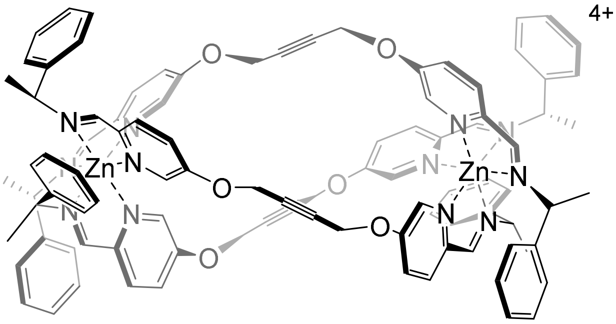

Λ_{Zn}-[Zn_2**L**^{2d}$_3$][ClO_4]$_4$ was synthesised using the procedure described for Λ_{Zn}-[Zn_2**L**^{2b}$_3$][ClO_4]$_4$, substituting 5,5'-(2,2'-oxybis(ethane-2,1-diyl)bis(oxy))dipicolinaldehyde for 5,5'-(but-2-yne-1,4-diylbis (oxy))dipicolinaldehyde.

Yield 0.152 g, 38%.

^{1}H NMR (400 MHz, 298 K, CD_3CN) δ_H 8.05 (6H, s, CHN), 7.48 (6H, dd, $^3J_{HH}$ = 8.0 Hz, $^4J_{HH}$ = 2.5 Hz), 7.40 (6H, dd, $^3J_{HH}$ = 8.0 Hz, $^4J_{HH}$ = 2.5 Hz), 7.08 (6H, t, $^3J_{HH}$ = 7.0 Hz), 7.04 (6H ,d, $^3J_{HH}$ = 2.5 Hz), 6.94 (12H, t, $^3J_{HH}$ = 8.0 Hz), 6.65 (12H, d, $^3J_{HH}$ = 7.0 Hz, Ar), 5.39 (6H, q, $^3J_{HH}$ = 6.0 Hz, CH), 4.88 (12H, s, CH_2), 1.58 (18H, d, $^3J_{HH}$ = 6.0 Hz, CH_3).

^{13}C{^{1}H} NMR (101 MHz, 298 K, CD_3CN) δ_C 161.7 (CHN), 142.0, 140.4, 140.1, 139.7, 132.3, 129.7, 128.6, 126.6, 123.1 (Ar), 82.7 (C) 64.9 (CH), 58.5 (CH_2), 23.6 (CH_3).

MS (ESI) m/z 410 [Zn_2L_3]$^{4+}$.

IR υ cm^{-1} 2971 m, 1567 w, 1225 m, 1076 s, 621 m.

Elemental analysis found (calculated for $C_{96}H_{90}Cl_4N_{12}O_{22}Zn_2.6H_2O$) % C 53.56 (53.77), H 4.28 (4.79), N 7.72 (7.84).

Λ_{Zn}-[Zn$_2$L^{2e}$_3$][ClO$_4$]$_4$

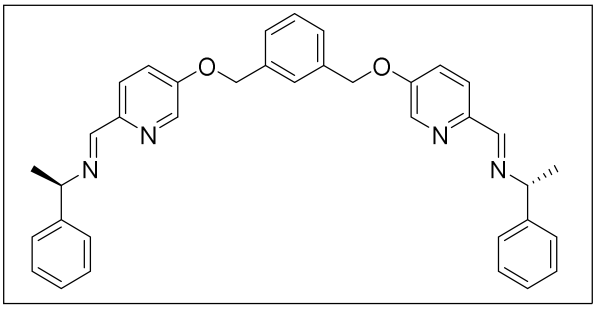

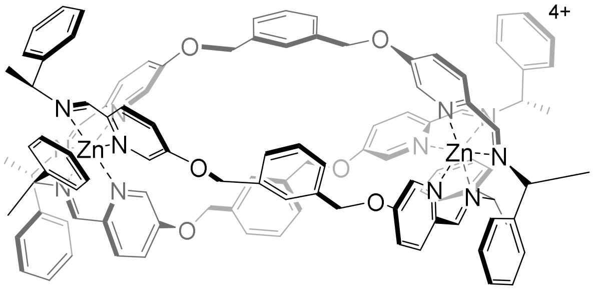

Λ_{Zn}-[Zn$_2$**L^{2e}**$_3$][ClO$_4$]$_4$ was synthesised using the procedure described for Λ_{Zn}-[Zn$_2$**L^{2b}**$_3$][ClO$_4$]$_4$, substituting 5,5'-(2,2'-oxybis(ethane-2,1-diyl)bis(oxy))dipicolinaldehyde for 5,5'-(1,3-phenylenebis (methylene))bis(oxy)dipicolinaldehyde.

Yield 0.220 g, 55%.

MS (ESI) m/z 502 [L+H]$^+$, 409 [Zn$_2$L$_3$]$^{4+}$.

IR υ cm^{-1} 2972 w, 1569 m, 1225 m, 1083 s, 702 m, 622 m.

Elemental analysis found (calculated for $C_{108}H_{102}Cl_4N_{12}O_{22}Zn_2.4H_2O$) % C 57.33 (57.28), H 4.41 (4.90), N 7.32 (7.40).

Λ_{Zn}-[Zn$_2$L^{2f}$_3$][ClO$_4$]$_4$

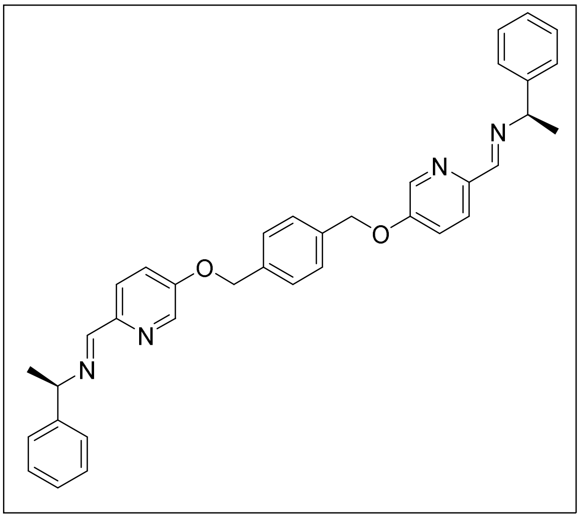

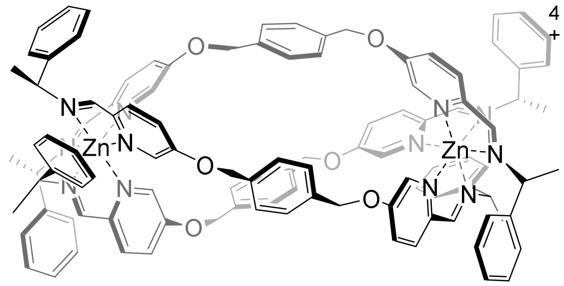

Λ_{Zn}-[Zn$_2$**L^{2f}**$_3$][ClO$_4$]$_4$ was synthesised using the procedure described for Λ_{Zn}-[Zn$_2$**L^{2b}**$_3$][ClO$_4$]$_4$, substituting 5,5'-(2,2'-oxybis(ethane-2,1-diyl)bis(oxy))dipicolinaldehyde for 5,5'-(1,4-phenylenebis (methylene))bis (oxy)dipicolinaldehyde.

Yield 0.051 g, 24%.

^1H NMR (400 MHz, , 298 K, CD_3CN) δ_H 8.15 (6H, s, CHN), 7.65 (6H, dd, $^3J_{HH}$ = 8.5 Hz, $^4J_{HH}$ = 3.0 Hz), 7.47 (24H, s), 7.44 (6H, d, $^3J_{HH}$ = 8.5 Hz), 7.31 (6H, d, $^3J_{HH}$ = 2.5 Hz), 7.16 (6H, t, $^3J_{HH}$ = 2.5 Hz), 7.03 (12H, t, $^3J_{HH}$ = 7.5 Hz), 6.71 (12H, d, $^3J_{HH}$ = 8.0 Hz, Ar), 5.46 (6H, q, $^3J_{HH}$ = 6.5 Hz, CH), 1.67 (18H, d, $^3J_{HH}$ = 7.0 Hz, CH_3).

$^{13}C\{^1H\}$ NMR (101 MHz, 298 K, CD_3CN) δ_c 162.0 (CHN), 160.6, 142.5, 140.5, 138.5, 137.0, 132.5, 130.9, 129.6, 129.0, 128.5, 126.3, 125.3 (Ar), 72.9 (CH_2), 65.3 (CH_3), 24.8 (CH).

MS (ESI) m/z 502 $[L+H]^+$, 409 $[Zn_2L_3]^{4+}$.

IR υ cm^{-1} 2974 w, 1569 m, 1224 m, 1083 s, 702 m, 651 m.

Elemental analysis found (calculated for $C_{108}H_{102}Cl_4N_{12}O_{22}Zn_2.4H_2O$) % C 56.79 (57.28), H 4.30 (4.90), N 7.32 (7.40).

Water soluble iron(II) flexicates[5]

Λ_{Fe}-$[Fe_2L^{2b}_3]Cl_4$

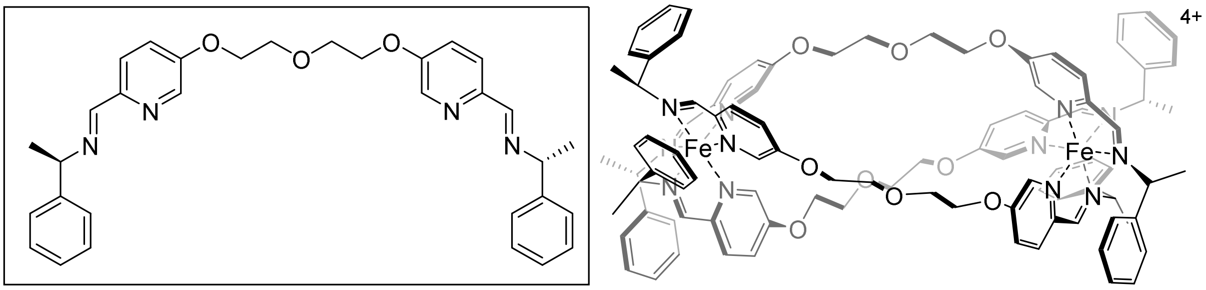

5,5'-(2,2'-oxybis(ethane-2,1-diyl)bis(oxy))dipicolinaldehyde (0.1 g, 0.32 mmol) and (*R*)-1-phenylethan-1-amine (0.08 g, 0.63 mmol) were dissolved in methanol. Iron (II) chloride (0.03 g, 0.21 mol) was added and an immediate colour change to deep purple was seen. The solution was heated to reflux (75°C) for 48 h. The solvent was removed under reduced pressure to yield a dark purple solid.

Yield 0.235 g, 78%.

^1H NMR (400 MHz, 298 K, MeOD) δ_H 8.80 (6H, s, CHN), 7.47 (6H, br s), 7.13 (6H, t, $^3J_{HH}$ = 7.5 Hz), 7.04 (12H, t, $^3J_{HH}$ = 7.5 Hz), 6.64 (12H, d, $^3J_{HH}$ = 7.5 Hz), 6.47 (6H, s), 6.05 (6H, s, Ar), 5.26 (6H, q, $^3J_{HH}$ = 6.5 Hz, CH), 4.65 (12H, br s), 4.59 (12H, br s, CH_2), 1.99 (18H, d, $^3J_{HH}$ = 6.5 Hz, CH_3).

$^{13}C\{^1H\}$ NMR (101 MHz, 298 K, MeOD) δ_C 171.2 (CHN), 159.0, 152.4, 145.0, 141.7, 131.4, 130.3, 130.0, 128.7, 125.7, 121.2 (Ar), 70.3 (CH_3), 69.8, 26.3 (CH_2).

MS (ESI) m/z 420 $[Fe_2L_3]^{4+}$, 522 [L+H].

IR υ cm^{-1} 3373 br s, 2927 s, 1557 s, 1491 m, 1450 m, 1300 m, 1231 s, 1122 m, 1038 s, 921 w, 841 w, 760 m, 660 m.

Elemental analysis found (calculated for $C_{96}H_{102}Cl_4Fe_2N_{12}O_9 14H_2O$) % C 55.03 (55.61), H 5.81 (6.32), N 7.74 (8.11).

Δ_{Fe}-[Fe$_2$L^{2b}$_3$]Cl$_4$

Δ_{Fe}-[Fe$_2$**L**^{2b}$_3$]Cl$_4$ was synthesised using the procedure described for Λ_{Fe}-[Fe$_2$**L**^{2b}$_3$]Cl$_4$, substituting (*R*)-1-phenylethan-1-amine for (*S*)-1-phenylethan-1-amine.

Yield 0.271g, 90%.

MS (ESI) m/z 420 $[Fe_2L_3]^{4+}$, 522 [L+H].

IR υ cm^{-1}: 3366 br s, 2987 m, 1556 s, 1486 m, 1450 m, 1382 w, 1231 s, 1121 m, 1039 m, 924 w, 842 w, 761 m, 700 m, 543 m.

Elemental analysis found (calculated for $C_{96}H_{102}Cl_4Fe_2N_{12}O_9.13H_2O$) % C 56.26 (56.09), H 5.95 (6.28), 7.99 (8.18).

Λ_{Fe}-[Fe$_2$L^{2c}$_3$]Cl$_4$

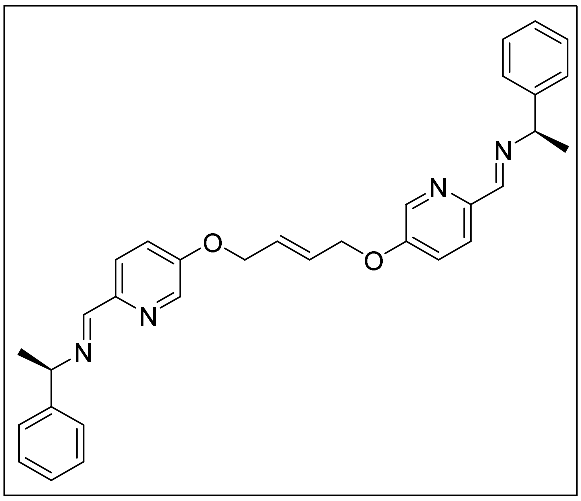

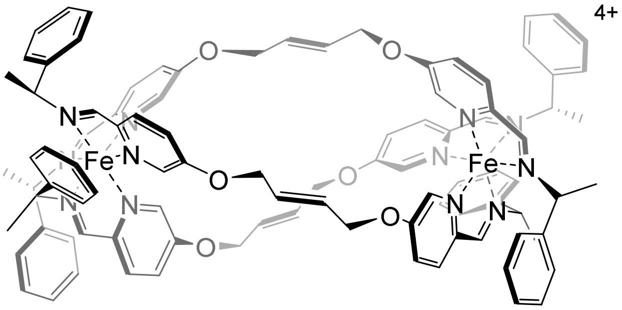

Λ_{Fe}-[Fe$_2$**L**^{2c}$_3$]Cl$_4$ was synthesised using the procedure described for Λ_{Fe}-[Fe$_2$**L**^{2b}$_3$]Cl$_4$, substituting 5,5'-(2,2'-oxybis(ethane-2,1-diyl)bis(oxy))dipicolinaldehyde for (E)-5,5'-(but-2-ene-1,4-diylbis(oxy))dipicolin aldehyde.

Yield 0.388 g, 97%.

^{1}H NMR (400 MHz, 298 K, MeOD) δ_H 8.80 (6H, s, CHN), 7.47 (6H, s), 7.13 (6H, t, $^3J_{HH}$ = 7.0 Hz), 7.04 (12H, t, $^3J_{HH}$ = 7.0 Hz), 6.64 (12H, d, $^3J_{HH}$ = 7.0 Hz), 6.44 (6H, s), 6.05 (6H, s, Ar), 5.26 (6H, q, $^3J_{HH}$ = 6.0 Hz, CH), 4.65 (12H, s, CH$_2$), 4.59 (6H, br s, CH), 1.99 (18H, d, $^3J_{HH}$ = 6.0 Hz, CH$_3$).

[For ^{1}H and ^{13}C NMR plots see manuscript]

$^{13}C\{^{1}H\}$ NMR (101 MHz, 298 K, MeOD) δ_C 171.0 (CHN), 158.8, 152.5, 144.8, 141.7, 131.2, 130.1, 129.8, 128.5, 125.5 (Ar), 121.0 (CH), 70.0 (CH_2), 69.6 (CH), 26.1 (CH_3).

MS (ESI) m/z 406 $[Fe_2L_3]^{4+}$, 505 [L+H].

IR υ cm^{-1} 3352 br s, 2970 br s, 1589 s, 1557 w, 1488 w, 1381 m, 1299 s, 1067 m, 1028 m, 760 m, 699 s, 562 w.

Elemental analysis found (calculated for $C_{96}H_{96}Cl_4Fe_2N_{12}O_6.9H_2O$) % C 59.92 (59.76), H 5.84 (5.96), N 8.63 (8.71).

Δ_{Fe}-$[Fe_2L^{2c}_3]Cl_4$

Δ_{Fe}-$[Fe_2\mathbf{L}^{\mathbf{2c}}_3]Cl_4$ was synthesised using the procedure described for Λ_{Fe}-$[Fe_2\mathbf{L}^{\mathbf{2c}}_3]Cl_4$, substituting (*R*)-1-phenylethan-1-amine for (*S*)-1-phenylethan-1-amine.

Yield 0.340 g, 89%.

MS (ESI) m/z 406 $[Fe_2L_3]^{4+}$, 505 [L+H].

IR υ cm^{-1} 3360 br s, 2928 m, 1589 w, 1557 s, 1485 m, 1451 m, 1381 w, 1299 m, 1230 s, 1135 w, 1069 m, 976 s, 836 w, 760 m, 699 m, 545 m.

Elemental analysis found (calculated for $C_{96}H_{96}Cl_4Fe_2N_{12}O_6.9H_2O$) % C 59.34 (59.76), H 5.93 (5.96), N 8.54 (8.71).

Λ_{Fe}-$[Fe_2L^{2d}_3]Cl_4$

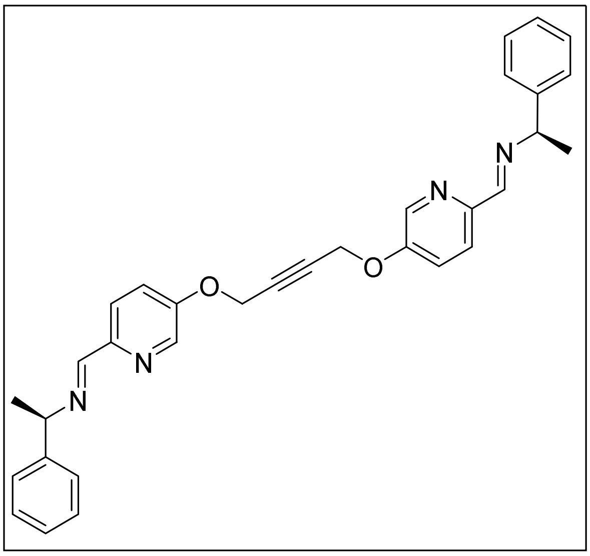

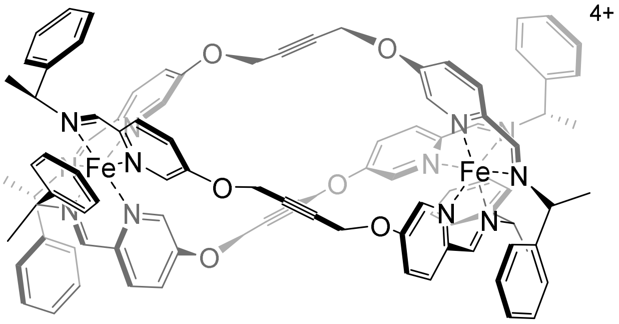

Λ_{Fe}-$[Fe_2\mathbf{L}^{\mathbf{2d}}_3]Cl_4$ was synthesised using the procedure described for Λ_{Fe}-$[Fe_2\mathbf{L}^{\mathbf{2b}}_3]Cl_4$, substituting 5,5'-(2,2'-oxybis(ethane-2,1-diyl)bis(oxy))dipicolinaldehyde for 5,5'-(but-2-yne-1,4-diylbis(oxy))dipicolin aldehyde.

Yield 0.301 g, 86%.

^{1}H NMR (400 MHz, 298 K, MeOD) δ_H 8.83 (6H, s, CHN), 7.57 (6H, t, $^{4}J_{HH}$ = 2.5 Hz), 7.47 (6H, d, $^{3}J_{HH}$ = 4.5 Hz), 7.12 (6H, t, $^{3}J_{HH}$ = 7.0 Hz), 7.01 (12H, t, $^{3}J_{HH}$ = 7.0 Hz), 6.66 (12H, d, $^{3}J_{HH}$ = 7.0 Hz), 6.27 (6H, d, $^{4}J_{HH}$ = 2.5 Hz, Ar), 5.34 (6H, q, $^{3}J_{HH}$ = 6.5 Hz, CH), 4.90 (12H, s, CH_2), 2.01 (18H, d, $^{3}J_{HH}$ = 6.5 Hz).

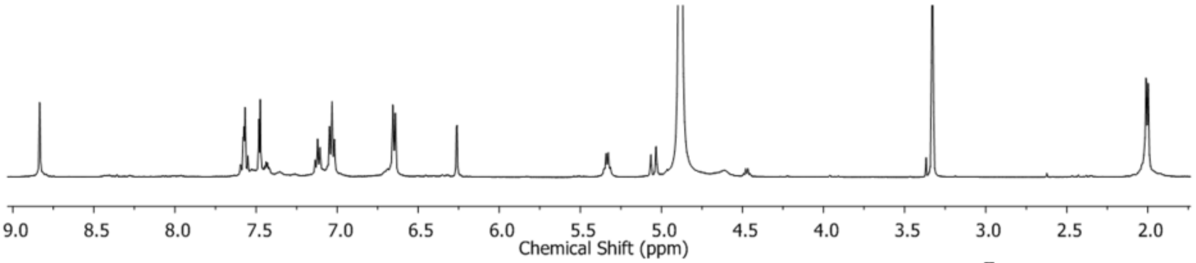

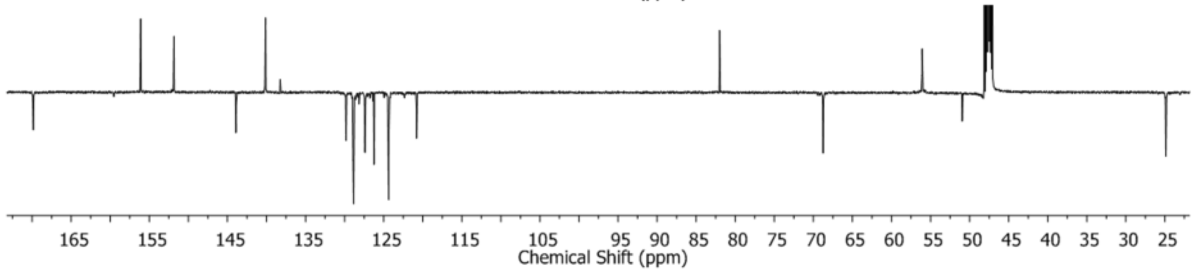

^{13}C{^{1}H} NMR (101 MHz, 298 K, MeOD) δ_C 157.7 (Ar), 153.2 (CHN), 145.3, 141.7, 131.2, 130.3, 128.8, 127.6, 125.8, 122.2 (Ar), 83.4 (C≡C), 70.2 (CH), 57.5 (CH_2), 26.3 (CH_3).

MS (ESI) m/z 405 $[Fe_2L_3]^{4+}$, 503 [L+H].

IR υ cm^{-1} 3350 br, s, 2926 m, 1557 m, 1490 m, 1223 s, 987 s, 836 w, 780 m, 699 m, 535 w.

Elemental analysis found (calculated for $C_{96}H_{90}Cl_4Fe_2N_{12}O_6.9H_2O$) % C 59.62 (59.95), H 5.45 (5.66), N 8.56 (8.74).

Δ_{Fe}-[$Fe_2$$L^{2d}_3$]$Cl_4$

Δ_{Fe}-[Fe_2**L**$^{2d}_3$]Cl_4 was synthesised using the procedure described for Λ_{Fe}-[Fe_2**L**$^{2d}_3$]Cl_4, substituting (*R*)-1-phenylethan-1-amine for (*S*)-1-phenylethan-1-amine.

Yield 0.319 g, 91%.

^{1}H NMR spectra (400 MHz, MeOD) were complicated by the presence of several conformational isomers (see Fig. 3 in manuscript) but variable temperature behaviour was analogous to that of the Zn(II) complexes. Between 213 and 313 K the phenethylamine methyl group (1.5 - 2 ppm) and imine (8.6 – 9 ppm) regions (Fig. S4) contain resonances consistent with the presence of two species – one of high-symmetry and one low – in thermodynamic equilibrium (ratio ca 1:0.75 at low temperature, increasing to 1:1.2 at high temperature).

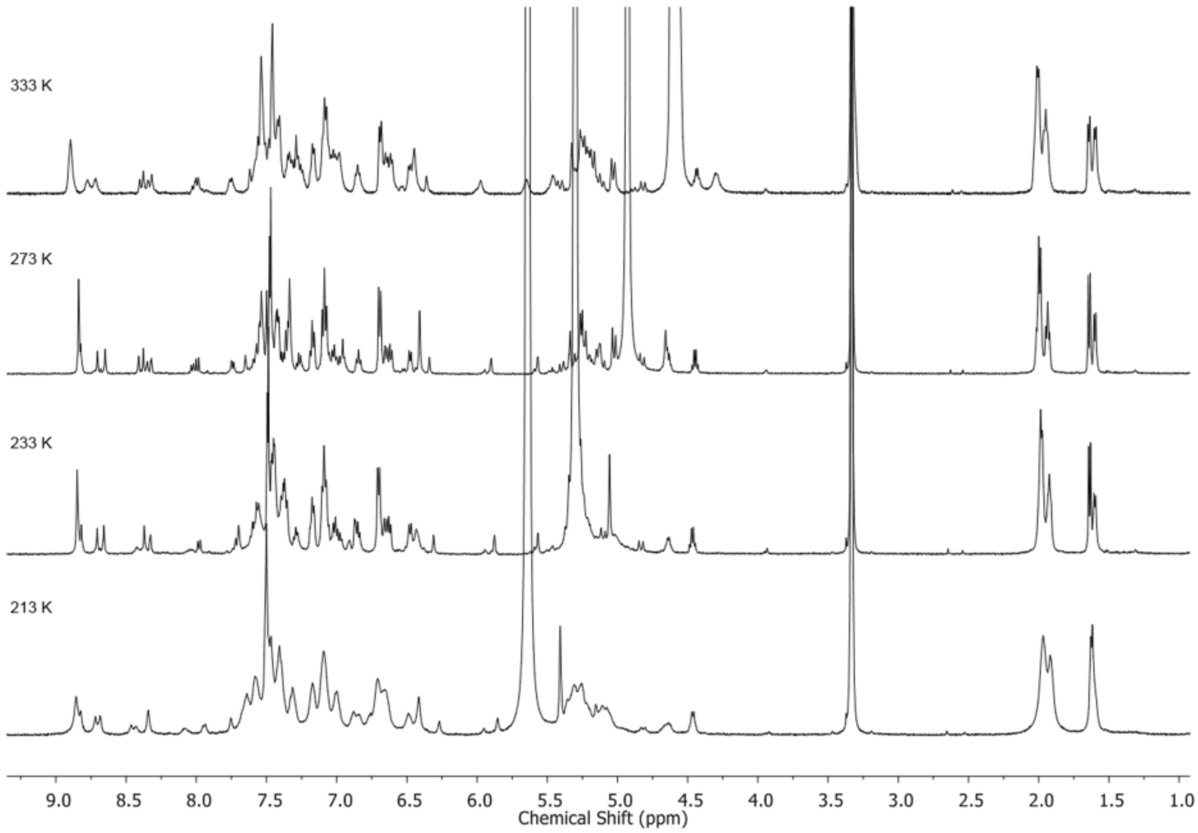

Figure S4. ^1H NMR spectra of Λ_{Fe}-[Fe_2**L**$^{2e}{}_3$]Cl_4.9H_2O between 213 K and 333 K in d^4-methanol.

MS (ESI) m/z 405 [Fe_2L_3]$^{4+}$, 503 [L+H].

IR υ cm^{-1} 3362 br s, 2928 br s, 1557 m, 1488 w, 1449 w, 1298 m, 1224 s, 988 m, 759 m, 699 s, 533 w, 467 w.

Elemental analysis found (calculated for $C_{96}H_{90}Cl_4Fe_2N_{12}O_6$.8$H_2O$) % C 60.64 (60.51), H 5.49 (5.61), N 8.82 (8.82).

Λ_{Fe}-[$Fe_2L^{2e}{}_3$]Cl_4

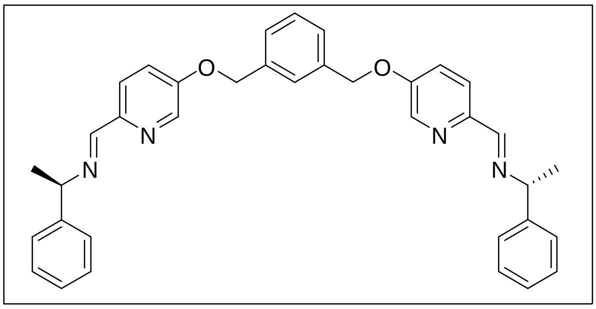

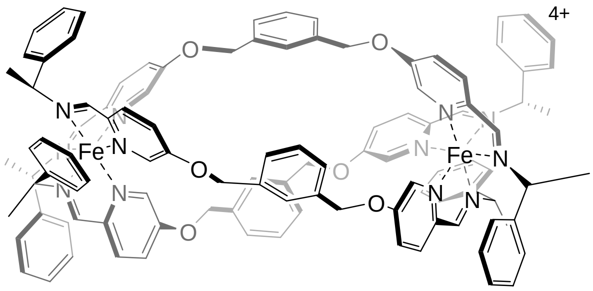

Λ_{Fe}-[Fe_2**L**$^{2e}{}_3$]Cl_4 was synthesised using the procedure described for Λ_{Fe}-[Fe_2**L**$^{2b}{}_3$]Cl_4, substituting 5,5'-(2,2'-oxybis(ethane-2,1-diyl)bis(oxy))dipicolinaldehyde for 5,5'-(1,3-phenylenebis (methylene))bis(oxy) dipicolinaldehyde.

Yield 0.228 g, 76%.

MS (ESI) m/z 444 $[Fe_2L_3]^{4+}$

IR υ cm^{-1} 3352 br s, 3026 br s, 1556 m, 1491 m, 1299 m, 1230 s, 1069 w, 997 m, 759 m, 698 s, 532 w.

Elemental analysis found (calculated for $C_{108}H_{102}Cl_4Fe_2N_{12}O_6.7H_2O$) % C 63.62 (63.47), H 5.73 (5.72), N 7.96 (8.22).

Δ_{Fe}-[Fe$_2$L^{2e}$_3$]Cl$_4$

Δ_{Fe}-[Fe_2**L**^{2e}$_3$]Cl_4 was synthesised using the procedure described for Λ_{Fe}-[Fe_2**L**^{2e}$_3$]Cl_4, substituting (*R*)-1-phenylethan-1-amine for (*S*)-1-phenylethan-1-amine.

Yield 0.198 g, 66%.

MS (ESI) m/z 444 $[Fe_2L_3]^{4+}$

IR υ cm^{-1} 3366 br s, 2970 br s, 1557 m, 1491 w, 1226 s, 1082 w, 1010 m, 759 m, 698 s, 531 w.

Elemental analysis found (calculated for $C_{108}H_{102}Cl_4Fe_2N_{12}O_6.3H_2O$) % C 65.99 (65.79), H 5.67 (5.52), N 8.53 (8.53).

Biophysical analysis of iron(II) flexicates

Cirular dichroism

Each compound was dissolved in water to 0.03 mM and spectra were measured on a Jasco J-815 spectrometer, calibrated conventionally using a 0.060% ACS holmium filter. Measurements were collected using a 1 cm path-length quartz cuvette. The parameters used were; bandwidth 1 nm, response time 1 sec, wavelength scan range 200 – 750 nm, data pitch 0.2 nm, scanning speed 100 nm/min and accumulation 4.

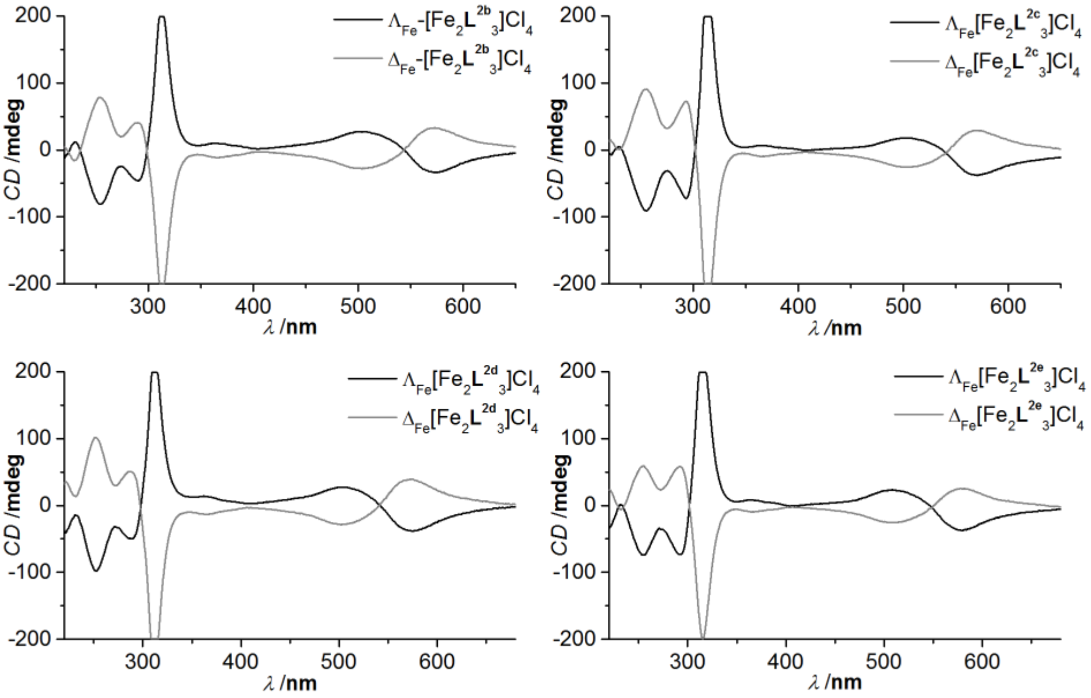

Figure S5. CD spectra of iron(II) flexicates (0.03 mM) in H_2O, showing each pair of enantiomers has equal and opposite spectra.

Thermogravimetric analysis

To determine the amount of water of crystallization present in the chloride salts of the flexicates – to compliment the elemental analysis data and IR spectra – thermogravimetric analysis was performed using a DSC1-1600 scanning calorimeter. An accurately weighed 40 µl aluminium crucible was heated from 298 to 650 K at 10 K/min under dinitrogen. The mass lost was plotted against temperature and the % mass loss from ambient temperature (1st plateau) to ~450 K (2nd plateau) was calculated. A worked example of Λ_{Fe}-[Fe_2**L**$^{\mathbf{2c}}$$_3$]$Cl_4$.$n$$H_2O$ is shown here (mass lost = 8.4%);

$$n = \frac{m_{\%} \times Mr_{anh}}{18(100 - m_{\%})}$$

$$\frac{8.4 \times 1766.50}{18(100 - 8.4)} = 9$$

n - stoichiometric equivalents of water

$m_{\%}$ - percentage mass loss

Mr_{anh} - anhydrous molecular weight

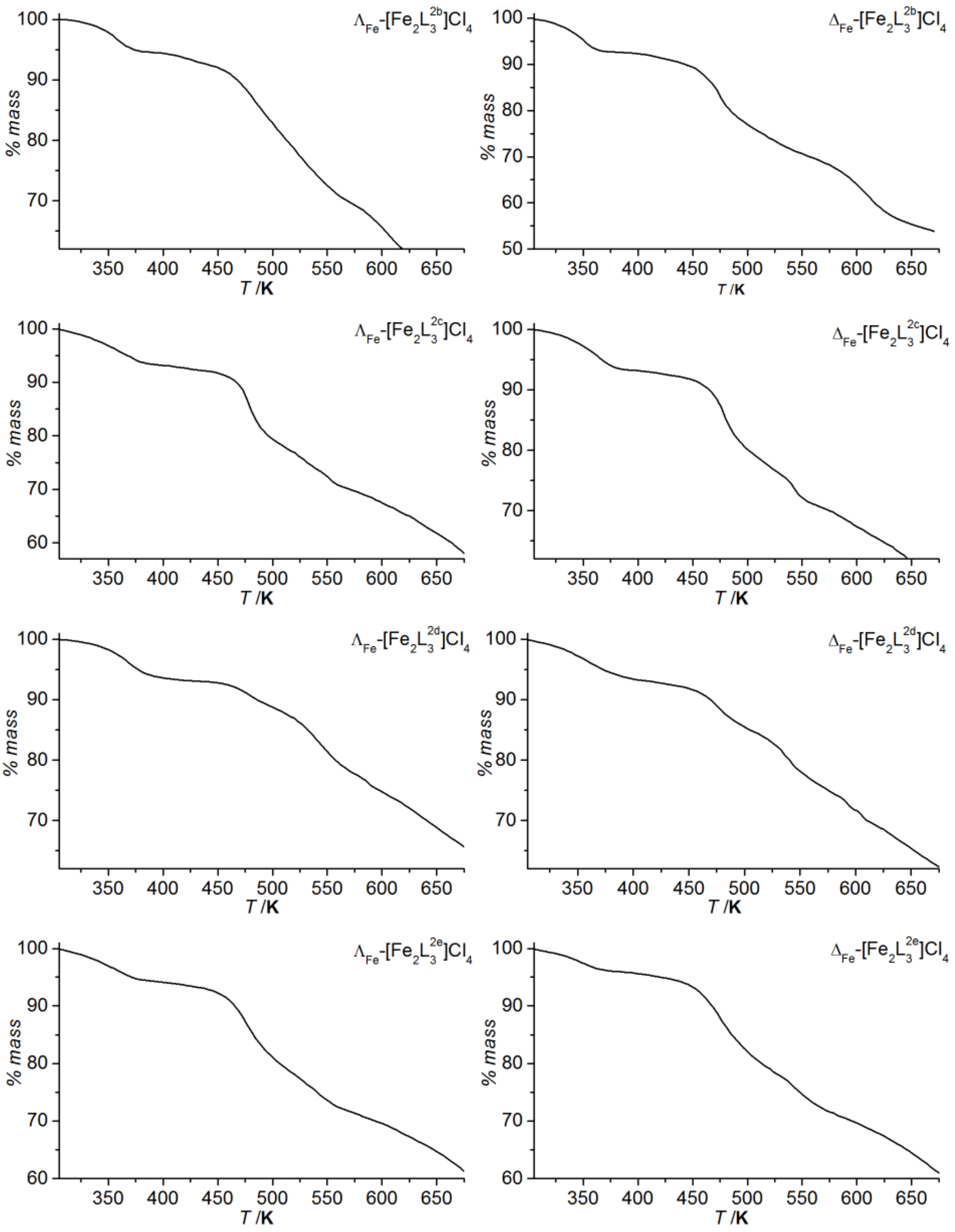

Figure S6. TGA spectra of iron(II) flexicates showing mass loss between 298 and 673 K.

Aqueous stability

Little decomposition of the flexicates occurred at pH 7 over a reasonable timescale, but half-lives for decomposition could readily be recorded in acid. Visible absorbance spectra for stability studies were recorded using a Carey IE spectrometer. Measurements were collected in a 1 cm path-length polystyrene cuvette and the standard parameters used were bandwidth 1 nm, response time 1 sec, wavelength scan range 350 – 800 nm, data pitch 0.2 nm, scanning speed 200 nm/min and accumulation 1. A 0.03 mM solution of each compound was measured in RPMI-1640 cell culture medium (37°C, every 6 h for 96 h) and 0.2 M hydrochloric acid (20°C, every 2 h for 24 h) was measured. The absorbance at 540 nm, which corresponds to the MLCT band of the complex, was used to quantify the concentration of the complex since peaks for decomposition products are unlikely to appear in this region. The complexes were observed to decay with first order kinetics. The half-lives ($t_{½}$) were calculated using the following equations;

$$\ln[A] = -kt + \ln[A]_0 \qquad t_{1/2} = \frac{\ln(2)}{k}$$

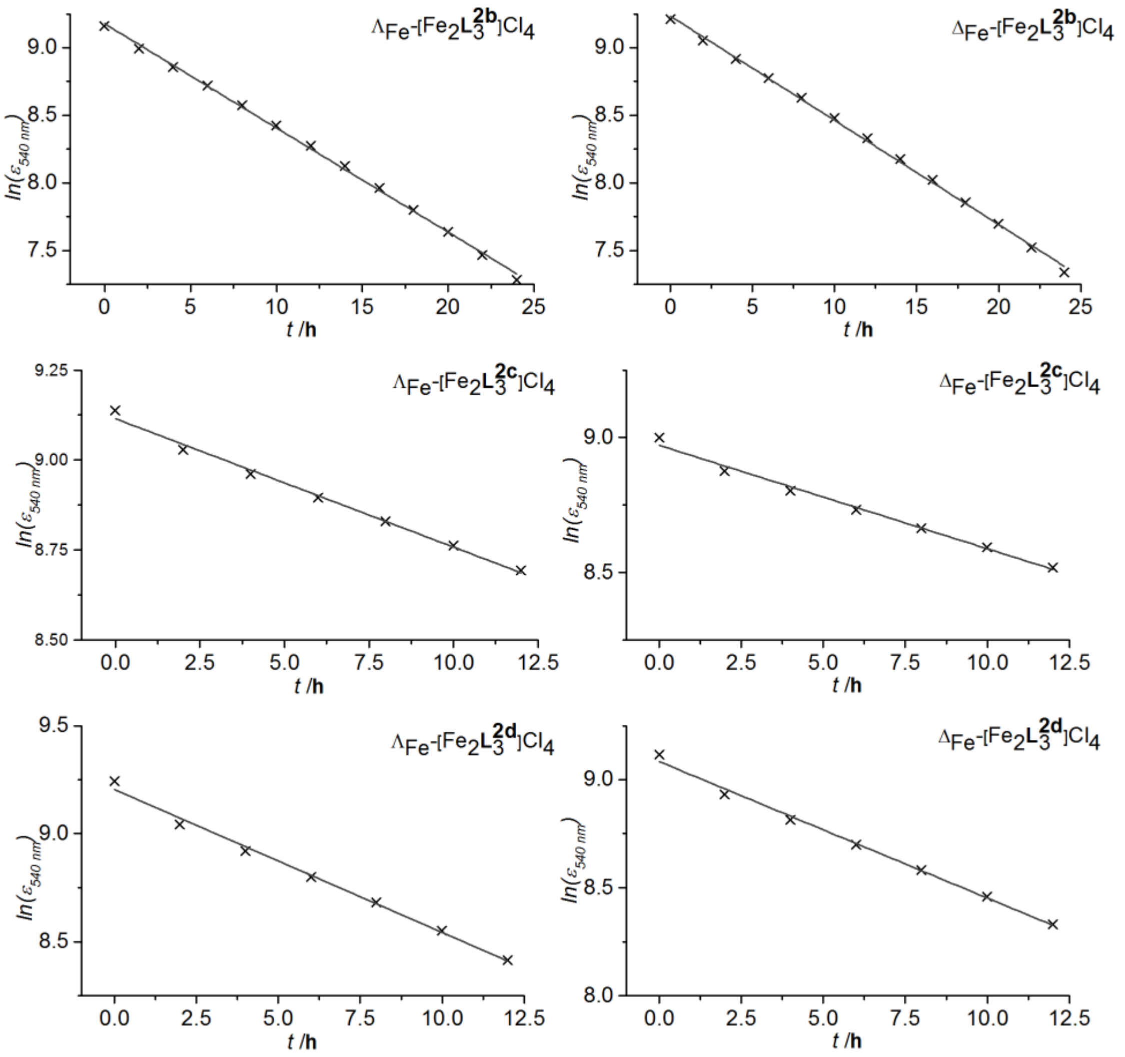

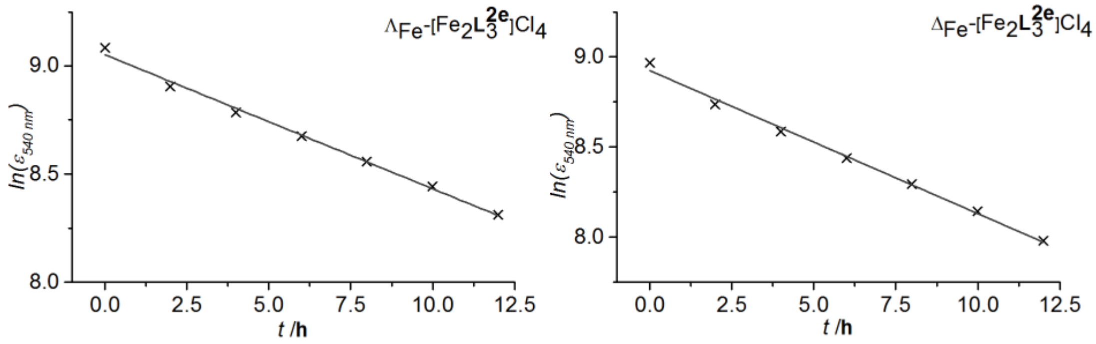

Figure S7. ln(ε) at 540 nm (corresponding to MLCT band) of iron(II) chloride flexicates and triplex metallohelices (0.03 mM in 0.2 M HCl).

Table S2. Solution half-life ($t_{½}$) of MLCT band (540 nm) for the iron (II) chloride flexicates, 0.03 mM in 0.2 M hydrochloric acid (pH 1.0) at 20°C.

Complex	$t_{½}$ /h (esd)
Δ_{Fe}-[$Fe_2L^{1a}_3$]Cl_4	9.6 (0.3)[5]
Λ_{Fe}-[$Fe_2L^{2b}_3$]Cl_4	9.0 (0.5)
Δ_{Fe}-[$Fe_2L^{2b}_3$]Cl_4	9.0 (0.5)
Λ_{Fe}-[$Fe_2L^{2c}_3$]Cl_4	19.5 (0.9)
Δ_{Fe}-[$Fe_2L^{2c}_3$]Cl_4	18.2 (0.5)
Λ_{Fe}-[$Fe_2L^{2d}_3$]Cl_4	10.5 (0.5)
Δ_{Fe}-[$Fe_2L^{2d}_3$]Cl_4	11.0 (0.5)
Λ_{Fe}-[$Fe_2L^{2e}_3$]Cl_4	11.2 (1.1)
Δ_{Fe}-[$Fe_2L^{2e}_3$]Cl_4	8.7 (0.9)

Antimicrobial activity

Verified stocks were used of Methicillin-resistant *Staphylococcus aureus*, USA300 JE2[28, 29] (MRSA) and *Escherichia coli*, TOP10 (*E. coli*).[30] Kanamycin[30] was used as a positive control.

MRSA and *E. coli* were grown to 10^8 cfu/ml (cfu = colony forming units) respectively in sterile Luria-Bertani (LB) medium, as measured by OD_{600} (optical density measured at wavelength: 600 nm) and confirmed by hemocytometer measurement. These were used to diluted of 10^6 cfu/ml in sterile Mueller-Hinton (MH) broth (15% w/v glycerol) and flash-frozen in liquid N_2 to store before use.

Minimum inhibitory concentrations (MICs)[6] were established using a macrobroth dilution method in cation-adjusted MH broth. 200 µl aliquots (128 µg/ml of each complex in sterilized MH broth, diluted 2^n µg/ml × 5) were added to 96-well plates in duplicate. This was inoculated with each bacterial strain (bacterial density of 10^3 cfu/ml, ~200 cells per well) and sealed. After mixing at 720 strokes/min for 10 seconds, growth was monitored over 20 h at 37 °C by recording OD_{600} every 10 mins with an iEMS 96-well plate reader. The lowest concentration to inhibit growth across each repeat is classified as the MIC. Positive (medium and untreated bacteria) and negative (medium only) controls were run with each plate.

Anticancer activity

Cancer cell lines were cultured in RPMI-1640 media supplemented with 10% foetal calf serum, sodium pyruvate (1 mM) and L-glutamine (2 mM). ARPE19 non-cancer cells–were cultured in DMEM/F12 (1:1), supplemented with 10% foetal calf serum and L-glutamine (2 mM) and–W138 cells were cultured in MEM (Gibco) supplemented with 10% fetal calf serum, L-glutamine (2mM), sodium pyruvate (1mM) and non-essential amino acids (0.1mM). Stocks of MDA-MB-468 (human breast adenocarcinoma), HCT116 $p53^{+/+}$ (human colon carcinoma, wild type p53), HCT116 $p53^{-/-}$ (human colon carcinoma, p53-null), ARPE19 (human retinal pigment epithelium) and W138 (human lung fibroblast) were maintained in T75 flasks in complete cell media. At 80% confluence cells were passaged and seeded into new T25 or T75 flasks or 96-well plates for chemosensitivity assays..

For determination of chemosensitivity, cells were seeded in 96-well plates at a cell concentration of 2.0 × 10^3 cells/well and incubated for 24 h at 37°C in an atmosphere of 5% CO_2, prior to drug exposure. All compounds were freshly dissolved in sterilized deionised water to give an initial concentration of 100 mM and diluted further with complete cell media to obtain concentrations ranging from 100 µM – 5 nM. Cell media (200 µl) was added to the reference cells and differing concentrations of drug solution (200 µl) were added to the remaining wells. The plates were incubated for a further 96 h at 37°C in an atmosphere of 5% CO_2. 3-(4,5-Dimethylthiazol-1-yl)-2,5-diphenyltetrazolium bromide (MTT) solution (0.5 mg/ml, 20 µl per well) was then added to each well and cells were incubated for a further 4 h at 37°C in an atmosphere of 5% CO_2. Upon completion all solutions were carefully removed from the wells and dimethyl sulfoxide (150 µl) was added to each well to dissolve any purple formazan crystals formed. A Thermo Scientific Multiskan EX microplate spectrophotometer was used to measure the absorbance at 540 nm. Lanes containing cell media only were used as a blank and untreated control cells (no drug)were used to calculate 100% cell survival. Cell survival for treated cells was determined as the absorbance of treated cells minus the blank, divided by the absorbance of the untreated control; this value was expressed as a percentage. The IC_{50} values were determined from a plot of percentage cell survival against drug concentration (µM). All assays were conducted in triplicate and the mean IC_{50} ± standard deviation was determined.

The Selectivity Index (*SI*) of each compound was determined by the IC_{50} in the healthy cell line ARPE19 divided by theIC_{50} of the same compound in the cancer cell line HCT116 $p53^{-/-}$.

Table S3. IC_{50} and *SI* values for all tested compounds in all cell lines.

Complex	MDA-MB-468 /µM (esd)	HCT116 $p53_{+/+}$ /µM (esd)	HCT116 $p53_{-/-}$ /µM (esd)	ARPE19/µM (esd)	W138 /µM (esd)	*SI*
cisplatin	2.44 (0.49)	3.51 (1.50)	8.12 (1.83)	3.43 (0.48)	2.17 (0.61)	0.42 (0.14)
Λ_{Fe}-[Fe_2**L**$^{2a}{}_3$]Cl_4	7.29 (0.29)	0.62 (0.08)	0.36 (0.04)	7.00 (0.82)	9.16 (0.80)	267 (64)
Δ_{Fe}-[Fe_2**L**$^{2a}{}_3$]Cl_4	8.36 (0.37)	0.87 (0.13)	0.43 (0.06)	12.06 (0.31)	4.69 (0.78)	154 (66)
Λ_{Fe}-[Fe_2**L**$^{2b}{}_3$]Cl_4	0.21 (0.10)	4.29 (1.85)	2.29 (0.32)	-		
Δ_{Fe}-[Fe_2**L**$^{2b}{}_3$]Cl_4	0.24 (0.07)	0.58 (0.25)	1.44 (0.60)	-		
Λ_{Fe}-[Fe_2**L**$^{2c}{}_3$]Cl_4	4.80 (3.27)	3.67 (0.54)	0.41 (0.17)	21.28 (5.47)	11.33 (3.08)	52 (30)
Δ_{Fe}-[Fe_2**L**$^{2c}{}_3$]Cl_4	3.59 (1.52)	0.36 (0.05)	0.04 (0.003)	33.42 (8.25)	12.64 (3.49)	836 (280)
Λ_{Fe}-[Fe_2**L**$^{2d}{}_3$]Cl_4	0.73 (0.22)	3.85 (0.94)	3.37 (1.21)			
Δ_{Fe}-[Fe_2**L**$^{2d}{}_3$]Cl_4	0.49 (0.01)	3.48 (0.19)	1.71 (0.77)			
Λ_{Fe}-[Fe_2**L**$^{2e}{}_3$]Cl_4	0.84 (0.06)	0.27 (0.03)	2.68 (0.49)	-		
Δ_{Fe}-[Fe_2**L**$^{2e}{}_3$]Cl_4	0.54 (0.002)	0.48 (0.04)	0.58 (0.18)	-		

Systemic and oral toxicity

Flexicates were dissolved in amoeba PYG growth culture medium before seeding with a 1:1000 (v/v) dilution of a fresh established amoeba culture. Amoeba cultures were then allowed to grow over 7 d and the impact on cell motility and replication (as measured by cell density) was assessed using an inverted microscope.

The flexicates were tested for oral and systemic toxicity using an insect metazoan animal model.[7] For the oral toxicity test cohorts of *Manduca sexta* one-day-old neonate larvae (n = 8) were fed on an artificial wheat germ diet laced with dilutions of test compounds. 25μg of each compound in sterile water was added to 1cm^3 food blocks and two larvae were placed onto each, with a total of 8 larvae per treatment. The caterpillars were allowed to feed for 7 days at 28°C and then weighed to assess growth rate. Results were expressed as average cohort weight and standard error and compared to a water control. Systemic toxicity assays were conducted essentially as previously reported for bacterial virulence assays.[8] Briefly, first day fifth instar *M. sexta* larvae raised on artificial wheat-germ diet were injected into an ethanol (70% v/v) swabbed region (using a 27-gauge insulin needle (VWR) just above the first proleg with a 100 µl of a dilution of the compounds (in 1xPBS). Caterpillars were placed on ice for 10 minutes prior to injection to reduce mobility. Cohorts of six larvae were injected with 50µg of each compound then allowed to continue feeding and placed at 28°C. They were monitored for feeding behaviour and normal development over 7 days, using physical stimulus to assess their status.

Denaturation of ct-DNA

ct-DNA (0.5 mg/ml, 7.5×10-5 per base, as determined by absorbance at 200 nm) was mixed with each complex (7.5 µM) in buffered conditions (10mM Tris, 1 mM EDTA at pH 7.0) to give 10 base: 1 complex. The absorbance at 260 nm as a function of temperature (every 1°C, 25-90°C) was measured in a 1 cm masked quartz cuvette at a rate of 0.4°C min-1 and run in triplicate. Tm was calculated from the first derivative of a Boltzmann sigmoidal fit of the plot of absorbance at 260 nm against temperature for each complex.

Induction of Apoptosis

Quantification of any cellular apoptosis and/or necrosis induced by the flexicates was performed as previously described.[10,11] In brief, HCT116 p53$^{+/+}$ cells were seeded at 5 × 10^5 cells/T25 flaskand incubated for 24 h at 37°C in 5% CO_2. Fresh media containing either no drug (control) or 20 µM flexicate was then added and cells were incubated for a further 48h before harvesting for apoptotic quantification. Adhered and floating cells were collected and pooled and following two PBS washes to remove residual media, cells were stained propidium iodide (100 µl) and Annexin-V-FLUOS labelling solution (Roche) in accordance with the manufacturer's instructions.The proportion of live, early apoptotic and late apoptotic/necrotic cells were then quantified by flow cytometry as previously.[10,11]

References

1. M. Seredyuk, A. B. Gaspar, V. Ksenofontov, Y. Galyametdinov, J. Kusz and P. Gütlich, *J. Am. Chem. Soc.*, 2008, **130**, 1431-1439.
2. S. M. Bakunova, S. A. Bakunov, T. Wenzler, T. Barszcz, K. A. Werbovetz, R. Brun and R. R. Tidwell, *J. Med. Chem.*, 2009, **52**, 4657-4667.
3. R. H. Mitchell and V. S. Iyer, *Synlett*, 1989, **1989**, 55-57.
4. R. Machinek and W. LÜTtke, *Synthesis*, 1975, **1975**, 255-256.
5. S. E. Howson, A. Bolhuis, V. Brabec, G. J. Clarkson, J. Malina, A. Rodger and P. Scott, *Nat. Chem.*, 2012, **4**, 31-36.
6. J. M. Andrews, *J. Antimicrob. Chemother.*, 2001, **48 (suppl 1)**, 5-16.
7. S. E. Reynolds, S. F. Nottingham and A. E. Stephens, *J. Insect Physiol.*, 1985, **31**, 119-127.
8. P. J. Daborn, N. Waterfield, C. P. Silva, C. P. Y. Au, S. Sharma and R. H. ffrench-Constant, *P. Natl. Acad. Sci.*, 2002, **99**, 10742-10747.
9. G. Koopman, C. Reutelingsperger, G. Kuijten, R. Keehnen, S. Pals and M. van Oers, *Blood*, 1994, **84**, 1415-1420.
10. S. J. Allison and J. Milner, *Cell Cycle*, 2007, **6**, 2669-2677.
11. S. J. Allison, J. R. P. Knight, C. Granchi, R. Rani, F. Minutolo, J. Milner and R. M. Phillips, *Oncogenesis*, 2014, **3**, e102.
